# Are feature assignment errors due to attraction? The case of Bulgarian numeral phrase

**DOI:** 10.3389/fpsyg.2025.1560012

**Published:** 2025-07-14

**Authors:** Danil Khristov, Penka Stateva, Julie Franck, Dávid György, Arthur Stepanov

**Affiliations:** ^1^Center for Cognitive Science of Language, University of Nova Gorica, Nova Gorica, Slovenia; ^2^Faculty of Psychology and Educational Sciences, University of Geneva, Geneva, Switzerland

**Keywords:** feature assignment, sentence processing, Bulgarian, agreement attraction, nominal concord

## Abstract

Well-studied attraction errors in speakers' production of subject-verb agreement arise from featural similarity between the attractor and the target (e. g., the verb) in the context of a specific syntactic relationship characterized by structural distance. This study examines production errors in a related but distinct type of featural manipulation: feature assignment in numeral phrases with modifiers such as “five rusty old windows,” in Bulgarian. The grammar of this language requires plural markers on the modifiers and a morphological count form on the final noun which speakers often erroneously replace with a regular plural form. In a series of four sentence completion experiments we demonstrate that speakers' errors in count form assignment are subject to linear rather than structural distance. Based on this, we argue that these errors are not due to attraction but instead reflect the cost of temporary storage and integration to resolve the assignment dependency, thus supporting linear distance-based theories of processing locality. Our findings also point to potential differences in the processing dynamics between agreement and feature assignment.

## 1 Introduction

Syntactic dependencies are paramount in natural languages and are a source of much of its expressive power. The principal mechanism by which natural language organizes syntactic dependencies involves some interaction of formal (morpho-) syntactic features across different parts of the sentence. Current psycholinguistic literature documents a significant body of research dedicated to processing of syntactic dependencies taking into account the featural perspective. The bulk of that research focused on agreement, a prototypical example of syntactic connectivity whereby the sentential subject and the verb share formal features such as person, gender and/or number. A particular interest has been on the mechanisms and routines underlying *agreement attraction errors*, the kind of errors speakers make associating the number and/or gender features of the verb with a nominal element (=“attractor”) other than the subject head, as in *The key to the cabinets #are lost*, in both production (Bock and Miller, 1991; Bock et al., 2001; Eberhard et al., 2005; Vigliocco and Nicol, 1998; Franck et al., 2002, 2006; Badecker and Kuminiak, 2007; Vigliocco and Franck, 1999, 2001) as well as comprehension (Pearlmutter et al., 1999; Wagers et al., 2009; Franck and Wagers, 2020, among others). The occurrence and relative rate of attraction errors have been attributed to a number of various influencing factors such as morphological or semantic similarity between the attractor and head (Wagers et al., 2009; Franck and Wagers, 2020; Smith et al., 2018, 2021), markedness of one feature value over another (Badecker and Kuminiak, 2007), grammatical as opposed to notional/conceptual source of the respective feature (Bock et al., 2001), as well as syntactic structure (see Section 2).

Building on the growing body of work on agreement attraction errors, in this study we extend the inquiry to *feature assignment*, a (morpho-)syntactic dependency distinct from, yet related to, agreement. Specifically, we examine numeral phrases (NumPs) such as five *old rusty windows* in Bulgarian, a Slavic language with rich nominal morphology which is under-represented in processing studies. Bulgarian numerals require a special morphological form, the so-called *count form*, on the head noun of the numeral phrase, which differs from the regular plural. However, speakers often default to a regular plural when one or more modifiers intervene, which raises the question of whether feature assignment triggers the same interference signatures as agreement, or whether its asymmetrical morphology engenders a different processing profile. By shifting the spotlight to processing below the clause level, inside the NP, and by comparing feature assignment with a related NP-internal syntactic dependency, nominal concord, we show across four production experiments that error rates rise with linear, but not structural, distance between the numeral and the noun. These findings reveal a kind of dependency that is sensitive to surface locality rather than hierarchical configuration, suggesting important processing differences between agreement and feature assignment and opening a new empirical window on processing sub-clausal morphosyntax at large.

## 2 Agreement attraction: a brief overview

Recent studies of agreement attraction converge on a consensus that attraction should be viewed in memory terms such as encoding and retrieval while adopting the content-addressable, cue-based approach to memory (e.g., McElree, 2000; McElree et al., 2003; Lewis and Vasishth, 2005). According to these models, agreement involves a cue-based memory retrieval of an agreement feature introduced in the preceding input during comprehension (Wagers et al., 2009) or production (Badecker and Kuminiak, 2007). In particular, agreement may be computed at the point of the verb via re-accessing the relevant features of the controller (e.g., the subject head) on the basis of similarity of respective morpho-lexical cues. Cue-based retrieval is sensitive to *interference* from other elements (encoding, as well as retrieval interference) temporarily stored and carrying syntactic features that are similar to those of the subject head. In line with the prediction of these memory models, studies conducted on various languages have reported a greater cost of processing of the agreement target when the intervener has the same case or agreement feature as the controller (e.g., Badecker and Kuminiak, 2007; Wagers et al., 2009; Franck and Wagers, 2020) or when it bears some semantic similarity to it (Smith et al., 2018). In the case of comprehension, attraction is often attributed to memory interference effects at retrieval (Wagers et al., 2009), in the case of production it is attributed to memory interference at encoding (Badecker and Kuminiak, 2007). However, Villata et al. (2018) argue that in addition to retrieval interference there's actually evidence in favor of encoding interference in comprehension as well. Their work suggests that what is typically interpreted as retrieval interference in comprehension can actually be effects that involve both encoding and retrieval interference.

Another important finding that came out of the agreement attraction research was that attraction errors are sensitive to the hierarchical position of the attractor with respect to the agreement controller or to the agreement target, in the syntactic tree. Four key pieces of evidence support this conclusion. First, Vigliocco and Nicol (1998) have shown that the ratio of agreement errors does not depend on the linear order “subject head followed by attractor followed by verb:” indeed the ratio of subject-verb agreement errors was found to be comparable in declarative sentences (e.g., ^*^the helicopter for the flights are safe) and corresponding yes-no questions where the verb precedes the subject (e.g., ^*^Are the helicopter for the flights safe?). Second, the hierarchical distance between the head of the subject phrase and the attractor was found to influence the rate of attraction errors. Franck et al. (2002) used subject NPs containing two stacked PPs, one embedded inside the other such as *The answer to the question(s) in the proposal(s)* and found that the plural on the noun within the higher PP (*questions*) elicited more attraction errors than the plural within the lower PP (*proposals*). Third, Bock and Cutting (1992) and Solomon and Pearlmutter (2004) have shown that the categorical status of the structural *domain* matters for attraction: an attractor embedded inside a relative clause induced less attraction effects than an attractor inside a PP (the linear distance between the subject head and the attractor was controlled for). Fourth, Franck et al. (2006) found attraction effects in structural domains beyond subject-verb agreement, in particular, with object clitics positioned in a particular hierarchical (c-commanding) position with respect to the verb, as predicted by transformational theories of syntax. The finding that production of agreement is constrained by structural factors is important as it signals involvement of mental grammar, in particular, its syntactic component, in the incremental production or comprehension of agreement.^1^ To sum up, the effects of structure-dependence and interference can be seen as staple characteristics of agreement attraction errors.

## 3 Feature assignment in Bulgarian nominal phrases

Bulgarian nouns belong to three grammatical genders (masculine, feminine, neuter) and are inflected for number; the specific inflection chosen is gender-dependent. In the context of a numeral, a dependent noun morphologically appears in a special count form which is homophonous with plural for feminine and neuter nouns and has a distinct ending for masculine ones:

(1) učebnik-Ø učebnic-**i** dva učebnik-**a**
textbook-sg textbooks-pl two textbooks-count
(2) kniga-Ø knig-**i** dve knig-**i**
book-sg books-sg two books- count (=pl)

Within the numeral phrase, any number of adjectival modifiers can be inserted between the numeral and the noun; these adjectivals always bear a (non-agreeing) plural form *- i* and can have a complex structure themselves.

(3) a. pet star-**i** prozore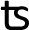-**a**
five old-pl window;m-m;count
‘five old windows'
b. pet star-**i** prašasal-**i** prozore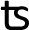-**a**
five old-pl dusty-pl window;m-m;count
‘five old dusty windows'

In spontaneous speech, speakers of Bulgarian tend to erroneously replace the count form with plural, producing instead instances such as (4):

(4) ^*^pet (star-**i**) (prašasal-**i**) prozor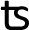-**i**
five old-pl dusty - pl window-pl
‘five old dusty windows'

This phenomenon has long been noted and documented by traditional and modern Bulgarian grammarians (Burov, 1989a,b; Pashov, 1989, p. 62; Peneva, 2021; see Barkalova et al., 2018 for a review). A growing body of diachronic and sociolinguistic research on this topic suggests that the Bulgarian count form tends to gradually yield to the regular plural as ongoing language change (cf. Haralampiev, 2006). The underlying dynamics and patterns of these errors in everyday language use have so far remained elusive, apart from the anecdotal observations that erroneous plural forms tend to be used with certain nouns more than with others (Peneva, 2021). Another idea occasionally entertained in the literature is that the errors have to do with the prescriptive character of the count form rule which is not observed across the board. While this may indeed account for some of the erroneous productions, this cannot account for the entire phenomenon as the ratio of errors was found to be dependent on a syntagmatic factor, namely, length of the feature assigning dependency. Stateva and Stepanov (2013) carried out a pilot production experiment which involved a cloze-like task in which participants had to complete a preamble ending with a numeral phrase whose final noun was missing and for which the lemma was provided (see description of our Experiments below). The number of intermediate adjectives was manipulated between zero and three. The results in this experiment showed the rate of production errors in Bulgarian numeral phrases linearly increases if more modifiers are added, so, for instance (3b) elicits more errors than (3a) which in turn elicits more errors than (1). Adding more linguistic material between the feature assigner (*pet*) and the feature recipient (*prozorets*-) in effect increases the distance or length of the feature assigning dependency. A follow up corpus study in Stateva and Stepanov (2016) fully replicated this effect.

**Figure 1:**
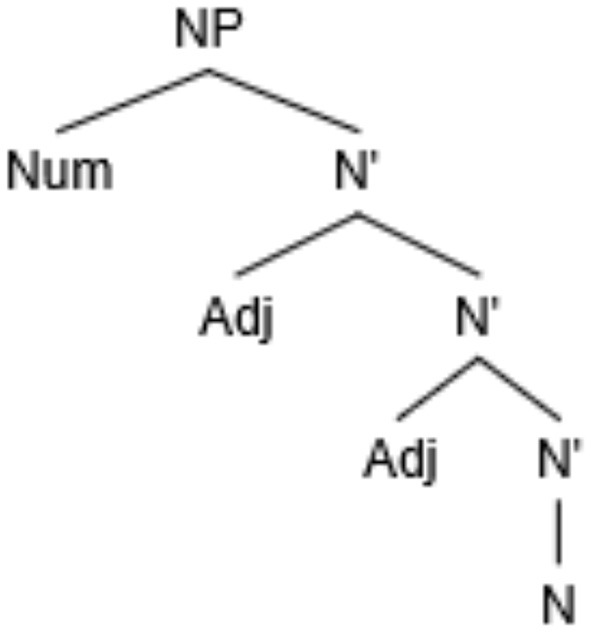
Syntactic structure of example (3b).

The distance between the numeral (Num) and noun (N) in (3b) can be formalized either in linear terms, that is, number of intervening lexical items (Adj) or in syntactic terms, e.g., number of intervening syntactic nodes (N'), which in both cases amount to 2 (Figure 1).

The previous studies aiming to differentiate between the two measures of distance in this construction led to somewhat divergent results. Stateva and Stepanov (2016) conducted an additional corpus search manipulating the number of syntactic nodes between one (an adjectival modified by an adverb) and two (two adjectives) while keeping the linear distance constant, comparing cases like (3b) and (5), in particular:

(5) pet [mnogo star-i] prozore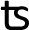-a
five very old-PL window;M-M;COUNT
‘five very old windows'

The authors reported that the number of errors in (5) is similar to the number of errors in (3a) and is significantly lower than (3b). These results argue against the role of linear distance in erroneous productions (since the number of words separating the two dependents does not capture the data) and for the relevance of the syntactic structure in modulating the ratio of errors [since the two conditions with similar errors, (5) and (3a), involved one syntactic node whereas condition (3b), which had more errors, involved two syntactic nodes]. On the other hand, Barkalova et al. (2018) compared structures (5) and (3a) in an experimental setting using a combination of a self-paced reading and sentence completion task similar to that used in Stateva and Stepanov (2013), while the number of lexical words and the number of plural forms between the numeral and the target noun were kept constant. The following are examples of the two conditions.

(6) trinajset predpotfian-i ot anglijsk-i-te
thirteen preferred-PLbyEnglish-PL-PL;DEF
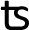
italel-i u
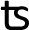
ebnik-a
reader;M-PL textbook;M-M;COUNT
‘thirteen textbooks preferred by English readers'
(7) trinajsetpredpotfian-iottfitalel-i
thirteenpreferred-PLbyreader;M-PL
anglijsk-iutfebnik-a
English-PLtextbook;M-M;COUNT
‘thirteen English textbooks preferred by readers'

The experiment failed to provide support for the role of syntactic structure in assigning the [+count] form as no difference in error production was found between the two conditions.

The observed discrepancy in the results could perhaps be attributed to differences in the tasks used, as the structural distance effect was found in the corpus study of written speech whereas the lack of effect was reported in real time production. In addition, in Barkalova et al. (2018)'s experiments the sentences were presented in their written form and participants had to complete the final noun by typing it. However, such a design gives the participant time to think about their answer, potentially introducing an unwanted rational bias in responding such as one based on the prescriptive rules pertaining to the conventional language norm. In the present study, we address this concern by employing, instead, the technique of oral elicitation of the final missing noun within a limited time frame.

## 4 Agreement vs. feature assignment

The count form on the final noun in the Bulgarian numeral phrase as in (1)-(2) represents an instance of *feature assignment*, a type of (morpho-)syntactic dependency that is related, but not identical, to agreement. In this construction, the feature assigner (the numeral) “dictates' a particular morphosyntactic feature on the (possibly non-local) noun, without bearing this form itself. For concreteness, we denote the relevant feature as [+count] by virtue of reflecting the countability property of the target noun (see e.g., Stepanov and Stateva, 2018; and references therein for discussion of this grammatical property in greater detail).

From the perspective of syntactic theory, feature assignment was seen as formally different from agreement in early generative transformational theories such as Government and Binding (Chomsky, 1981). In particular, the former was formalized via the notion of *government* whereas the latter was formalized in terms of a particular structural configuration, *specifier-head*. In the current minimalist framework, the two mechanisms of featural interaction are seen as largely part of the same syntactic process subject to feature valuation via a probe-goal searching algorithm termed *Agree* (Chomsky, 2001). Morphologically, agreement is a “symmetrical” phenomenon in that one and the same feature may be overtly represented on both ends of the sentential dependency (e.g., subject and verb). In other words, this relation is manifested in the morphological covariation between two parts of the dependency. In contrast, feature assignment is superficially more “asymmetrical,” in that the relevant morphological feature may be overtly represented only at the tail of the dependency, but not on its head. Given these noted similarities as well as differences between agreement and feature assignment, we may ask, therefore, whether the two dependency types also differ in terms of processing, in particular, in production.

The morphological asymmetry characteristic of feature assignment suggests a potentially different processing scenario for the latter compared to agreement insofar as morphological *cues* are concerned. The symmetric interaction of cues in agreement is reflected in the processing models in which the “communication” between the subject and the verb is captured in the context of mechanisms of working memory such as encoding of the relevant feature at the agreement controller, e.g., subject, and its cue-based retrieval at the agreement target, e.g., verb (see references above). In contrast, the asymmetric character of feature assignment suggests that no cue-based retrieval of the previously encoded feature of the controller takes place at the assignment target (the noun).^2^ Languages with rich nominal morphology where the assignment process can be tracked the easiest, may provide a particularly informative source of insight in studying the time course of feature assignment.

Nevertheless, progress in this domain so far has been limited. Reported studies up to date concentrated mostly on the effects of processing nominals that bear a particular feature. For instance, it has been argued that processing nominals marked with lexical case forms such as Dative, is associated with a greater difficulty than those marked with structural case such as Nominative or Accusative, in German (Bader and Bayer, 2006). This line of research also established that overt morphological case is used immediately and consistently to identify the agent and the patient/theme of a sentence (Bader and Meng, 1999, 2018). Another line of inquiry focused on the factors such as corpus frequency, mismatches between notional and grammatical gender or number may incur differential effects on lexical access (for instance, accessing one gender could be more demanding than other genders, cf. e.g., Slioussar and Malko, 2016). Yet another, related, line of work investigated priming effects of the adjectival modifier's features on the lexical access of the head noun (e.g., Gurjanov et al., 1985a,b). It remains largely unclear, however, how the feature recipient “communicates' with the feature assigner in non-local contexts and what processing mechanisms support this kind of dependency in real time, in comparison to agreement.

Therefore, we believe by comparing the processing aspects of feature assignment to those of the better-studied agreement constructions, we may gain superior insight as to similarities as well as differences between the two. Distinguishing between these two types of configurations is instructive in terms of better understanding the role of syntactic structure in featural manipulation in real time, the role of relevant memory representations and perhaps an even better insight as to whether they should be considered as two different grammatical processes or part of the same grammatical mechanism.

There is one other important peculiarity of numeral phrases involving modifying adjectives that needs to be taken into account. In effect, these phrases may involve not one, but two morphosyntactic dependencies. In the absence of a numeral, the plural noun agrees with the adjectival(s) as an instance of nominal concord, as discussed above. With the numeral in the picture, the noun is spelled out in the [+count] form dictated by the numeral; the accompanying adjectives still realize plural morphology through concord, as schematically shown in (8) (see Stepanov and Stateva, 2018 for syntactic details of the assignment of [+count] form in Slavic languages):

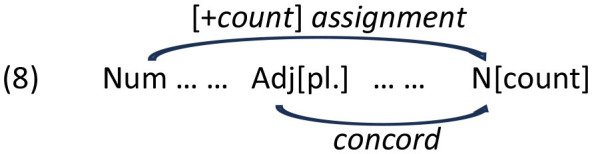

One may therefore ask if the observed distance effect in the error patterns found in Stateva and Stepanov's (2013, 2016) studies could actually stem not from distance but the number of intervening plural features on the adjectives, which covaries with distance. Activation-based models of memory such as ACT-R developed to account for sentence comprehension (Anderson et al., 2004; Lewis and Vasishth, 2005; Lewis et al., 2006) and their extension to structure building mechanisms in general, including production (Villata et al., 2018; Villata and Franck, 2019), provide a natural explanation for this observation, as each additional plural would come to reinforce the activation of the plural feature, thus increasing the risk of producing an erroneous plural feature on the target nouns. In line with this possibility, morphological priming studies using the Lexical Decision task, in which the adjectival modifier with the matching or non-matching agreement feature primed the target noun, demonstrated that nouns preceded by feature-matching adjectives were recognized faster than those preceded by feature-mismatching ones (Gurjanov et al., 1985a,b). This suggests that the repeated activation of a plural marker on an adjective may contribute to increasing the tendency to erroneously produce that marker on the following noun. If cumulative feature activation is the core factor at play in feature assignment, this factor may also explain observations by Barkalova et al. (2018)'s and Stateva and Stepanov's (2016) second corpus study who found no difference between conditions that involved the same number of intervening plural features [three for the former, cf. (6) vs. (7), and one for the latter, cf. (3a) vs. (5)]. The need to tease apart the various potential factors at play in the processing of feature assignment was part of the motivation for our present inquiry.

## 5 The present study

The question we ask in the present study is whether feature assignment errors in numeral phrases as in (4) can be treated on a par with agreement attraction errors, in other words, whether the two error types can be traced to the same or similar processing source(s). Our initial incentive for pitting the feature assignment in Bulgarian numeral phrases against the well-known agreement construction was the following: (i) both construction types elicit production errors which can be elicited in laboratory conditions; (ii) the ratio of errors in the numeral phrases reported in the works cited above (Section 3) was between 6%−12% in the simplest cases (without complex modifying material), which is comparable to error ratios reported in the agreement attraction literature; (iii) both effects occur at the tail of the respective dependency (verb in agreement, final noun in the numeral phrases), and, furthermore, (iv) both dependency types are subject to distance effects (see Franck et al., 2002). We reasoned that, if the errors speakers make in Bulgarian numeral phrases are triggered by the same kind of processing mechanisms or strategies as those in agreement, they should be sensitive to the same or similar set of factors or constraints influencing their production and comprehension discussed in the literature on agreement attraction. From this set, we focus on two perhaps most prominent and reliably established characteristics of the attraction phenomenon, namely, (i) the relevance of *syntactic structure* (see above) and (ii) *interference* or feature mismatch effect.

### 5.1 Syntactic structure

As we saw above, some crucial evidence for structural involvement in the agreement attraction literature is based on the structural distance effect (Franck et al., 2002). Distance is in principle a good indicator of processing complexity and, in relation to processing syntactic dependencies, it can be formalized either in structural or in linear terms (see also above). We believe sensitivity to structure can be viewed as a crucial litmus test for deciding one way or another precisely because it should indicate the degree of involvement of the grammatical, in particular, syntactic, component in the relevant production and/or comprehension process that these errors are a result of. Specifically, we approach the question whether structural consideration indeed modulates the ratio of errors in Bulgarian numeral phrases by asking whether the distance effect discussed in Section 2 follows as a result of degree of syntactic embedding. Alternatively, the distance effect can be simply linear, or in terms of number of lexical items intervening between the head (numeral) and tail (final noun) of a feature assignment dependency. It is not clear at this point which of the two conceptions of distance is relevant for Bulgarian numeral phrases. If the relevant measure of distance turns out to be structural, that would suggest that feature assignment errors in Bulgarian numeral phrases may have the same origin as agreement attraction errors and that, more generally, feature assignment follows a processing scenario that is at least in some important respects similar to agreement. This would be also in line with modern syntactic accounts (see above). Alternatively, if the relevant metric of measuring distance is only linear, this would suggest a process different from that operating in the agreement constructions.

### 5.2 Interference

If the errors as in (4) are indeed a result of attraction due to interference, what can possibly be an attractor/intervenor there? The erroneous plural marker on the final noun in (4) is the same as that on the modifying adjectivals so the latter are the obvious suspects. An important caveat in this case is that the putative attractor(s) are of a different syntactic category to the attraction target (final noun), though agreeing with it in number (plural). Attractors in the agreement attraction literature are generally of the same category (noun). So the question here is two-fold: (i) can the error in (4) be due to an interference of an unorthodox kind, namely, coming from an agreeing adjective, rather than within the same category (noun); (ii) do intervenors of the same category (noun) modulate the ratio of production errors in the Bulgarian numeral phrases? The answers to these questions will determine the relevance of the memory accounts of attraction involving the cue-based retrieval component.

In this study we report the results of three production experiments and one forced-choice sentence completion experiment aimed at exploration of feature assignment in Bulgarian numeral phrases in real time. Experiments 1–3 aim at elucidating the role of structural and linear distance. In particular, Experiment 1 focuses on the effect of linear distance whereas Experiments 2 and 3 test for the effect of structural distance on erroneous productions. For the first three experiments, we adopt the measures of linear and structural distance as in the corpus studies by Stateva and Stepanov (2016) experimental studies by Stateva and Stepanov (2013) and Barkalova et al. (2018). Specifically, linear (or Euclidean) distance is the distance measured between the numeral (the probe) and the target noun (the goal) in terms of intervening lexical items, whereas structural distance is the distance measured in terms of number of intermediate syntactic nodes (cf. Figure 1). Experiment 4 tests for potential interference effects affecting the pattern of production errors focusing on a number mismatch effect on erroneous productions in adjective-noun agreement where a plural target noun agrees with a preceding plural adjective. Since Experiment 3 focuses on the position of the intervenor (a designated adjective), rather than the numeral, relative to the final noun, the measure of distance in the fourth experiment is necessarily different from the other three: in particular, we follow Franck et al. (2002) in their measure of distance from the intervener to the target noun.

This study also addresses an additional, understudied dimension in research on processing featural dependencies. Most studies on agreement attraction phenomena, as reviewed above, focus on agreement at the clausal level—that is, the interaction between subject (or object) constituents and the verbal phrase. In memory models of attraction (cf. Lewis and Vasishth, 2005), a syntactic constituent, such as the clausal subject, is typically considered the target of a single encoding event, essentially following the X-bar theory of phrase structure (cf. Chomsky, 1981). The time course of processing featural dependencies at the sub-clausal level is rarely discussed in the processing literature; specifically, little is known about how phrasal heads are encoded in relation to their dependents and how featural interactions take place among them. A notable exception is Wagers and McElree (2022), who provide experimental evidence that familiar memory mechanisms of encoding and retrieval also apply to nominal concord, or noun-adjective agreement, in English (see Section 9.3). Nonetheless, a significant gap remains concerning the processing of featural dependencies directly within noun phrases (NPs). This study, therefore, seeks to expand our understanding of featural communication phenomena at the phrasal level, specifically within noun phrases. Experiments 1–3 address feature assignment, while Experiment 4 extends the investigation to nominal concord.

## 6 Experiment 1

Experiment 1 evaluates feature assignment in the numeral phrase in terms of length of the resulting dependency. In particular, we tested the hypothesis that linear distance (number of words) between the numeral and the target noun affects the rate of erroneous plural productions in assigning the count form on the noun in the Bulgarian numeral phrase. If feature assignment is sensitive to linear distance, as suggested by some of the previous studies, the rate of erroneous selection of the plural form is predicted to increase with linear distance. Importantly, the effect could not be accounted for by structural distance, since the number of intervening nodes between the numeral and the target noun was kept constant across conditions.

### 6.1 Method

#### 6.1.1 Participants

A total of 119 native adult Bulgarian speakers (90 females and 29 males, mean age = 44.42, SD = 12.57) participated in the experiment. They received no compensation for their participation.

#### 6.1.2 Materials

The materials consisted of 24 experimental items, each made up of a sentential preamble with a sentence-final subject in the form of a numeral phrase. The sentence-final numeral phrase contained a numeral, a modifying adjectival phrase, and was missing the final head noun. The lemma for the final noun was provided separately on the screen. For this experiment, target nouns were selected from the list of nouns with the highest rates of incorrect use in plural form after a numeral, in the Bulgarian National Corpus (Koeva et al., 2012). The following nouns have been excluded from this list:

- high frequency nouns in the extracted corpus data [*dni* (days), *p*γ*ti* (times)]f,
- the masculine noun form *p*γ*ti*(*times*) that mainly appears in a context with a numeral or a quantifier, but which is morphologically plural,
- animate nouns [*d*^*j*^*avol* (devil)],
- nouns that were natural dual (that come in groups of two naturally) [*lak*γ*t* (elbow)],
- nouns for which it was hard to construct a plural context [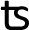
*ent*γ*r* (center)], and
- nouns which require a complement, and if used without one, would violate the theta criterion [*vid* (type, kind, species), *komponent* (component)].

Experimental items were constructed in three conditions, namely, Short, Medial, and Long. The intervening adjective phrase in the Short condition contained a single adjective or participle in the plural form modifying the target noun. In the Medial condition, it contained an adjective/participle modified by a PP that had a singular noun as a prepositional complement. In the Long condition, the adjective phrase included an adjective/participle modified by a PP that included a singular noun modified by another adjectival phrase which contained a head adjective modified by an adverb such as *mnogo “*very.” The NP within the PP was always in the singular. While all target nouns were masculine, half the intervening nouns were feminine and the other half neuter. Also, the number of plural markers in the intervening region was kept constant and restricted to one in all conditions. An example of the stimulus set in the three experimental conditions corresponding to the interpretations in (9) is illustrated in Table 1 (note that the word order in the respective Bulgarian sentences is OVS).

(9) **Sentence item translations by condition in Experiment 1**
Short: Seven recommended manuals proposed a solution to the problem.
Medial: Seven manuals recommended by the physician proposed a solution to the problem.
Long: Seven manuals recommended by the very famous physician proposed a solution to the problem.

**Table 1:** Example of a sentence item in Experiment 1.

**Preamble**	Reʃeni-e na problem-γ predlag-a-ha… solution;N-N;SG of problem;M-DEF.SG propose.IMPF 3; PL;PST	
Condition	Sentence-final NumP, modifier part	Target noun lemma
Short	sedem preporγtfan-i seven recommended-PL	narγtfnik- manual;M;SG (baseform)
Medial	sedem preporγtfan-i ot lekark-a-ta seven recommended-PL by physician;F-F;SG-DEF;F;SG	
Long	sedem preporγtfan-i ot mnogo izvestn-a-ta lekark-a seven recommended-PL by very famous-F;SG-DEF;F;SG physician;F-F;SG	

The grammatically correct form of the target noun in the experimental materials was always count. In order to hide this regularity, the experiment also contained 72 filler items with a structure similar to that of the experimental items. All fillers included sentences with a target noun in a (morphologically) plural form: 24 filler items whose target noun was feminine and modified by a numeral (the count form of feminine and neutral nouns, which is used when those nouns are modified by a numeral is morphologically identical to the plural form), 24 items whose final noun was masculine and not modified by a numeral, and 24 filler items whose target noun was feminine and not modified by a numeral. This ensured that half of the items (experimental or filler) had a numeral, and that half of the target nouns were masculine and the other half feminine. The numerals in the experimental and filler items were between two and *seven*. Each appeared an equal number of times.

The audio versions of the experimental stimuli were recorded in their entirety including the final noun in the grammatically correct form by a female native Bulgarian speaker (the last author) with a Zoom H4n Pro stereo recorder at a sampling rate of 44,100 Hz and 24-bit sampling depth. In each recording, the portion containing the final noun was then truncated using the Praat speech analysis software. This procedure was implemented in order to maximally preserve the natural intonational contour of the sentences during playback.

The materials were counterbalanced across participants. The items were distributed across three experimental lists such that each participant only saw one version of each experimental item. Each condition in the materials (both for target and filler items) was seen an equal number of times. Items were presented in a randomized order within each list.

#### 6.1.3 Procedure

The experiment was implemented on the online PCIbex platform (Zehr and Schwarz, 2018). After completing an online demographic questionnaire and consent form, participants listened to the preamble with a missing final noun through headphones or computer loudspeakers at a comfortable listening level (the decision not to pronounce the entire sentence but only the final target noun was made in order to reduce a potential fatigue effect). After the preamble was played back, a noun lemma appeared on the screen. At this point the participants were instructed to complete the preamble by speaking outloud the noun for which the lemma was provided, in the form they considered to be appropriate, and record their answer. Participants had to click on the start button on the screen in order to record the target noun. Once the target item was articulated, participants had to click on the stop before moving on to the next item. Prior to starting the experiment, participants were given two practice trials as well as an opportunity to test and listen to their voice in order to ensure their microphone was working. The practice trials consisted of a preamble and target nouns in masculine and feminine form. The preamble contained a count quantifier such as *njakolko “*certain” which requires a count form on the noun, similarly to numerals. In the masculine form, the special count morphology had to be used, whereas in the feminine form, the count form is homonymous with the regular plural (see above). The entire experiment lasted about 20–25 min.

#### 6.1.4 Data coding and analysis (Experiments 1, 2, and 4)

Participants' recordings were coded manually by a native speaker of Bulgarian (the first author). Accuracy was analyzed using Generalized Linear Mixed Effects Regression Models with the *lme4* package (Bates et al., 2015) in R (R Core Team, 2018). Main effects and interactions are reported from model comparisons where the minimal difference between two models is the target effect, while pairwise comparisons were obtained using the *emmeans* package.

All models contained Participant and Item as random effects. For all experiments, we report an initial hypothesis testing model containing only the target independent variable(s) as fixed effect(s). These analyses were then followed up with an exploratory model that included the Gender of the intervening noun as an additional fixed effect. Further exploratory analyses consisted of Spearman correlations between overall accuracy and sensitivity to intervention calculated as ACC_absent_-ACC_present_, as well as sensitivity to linear distance calculated in each case as pairwise differences between conditions. The full model summaries for all experiments can be found at the OSF repository (https://osf.io/8prhn/).

### 6.2 Results

No items showed performance that greatly differed from the overall distribution, so all experimental sentences were kept for analysis. Twenty-seven participants produced more plural forms than count forms overall (and therefore had < 50% correct responses), but given that this was the goal of the experimental manipulation, these participants were not removed. Two participants produced no count forms at all and were removed from analysis.

**Figure 2:**
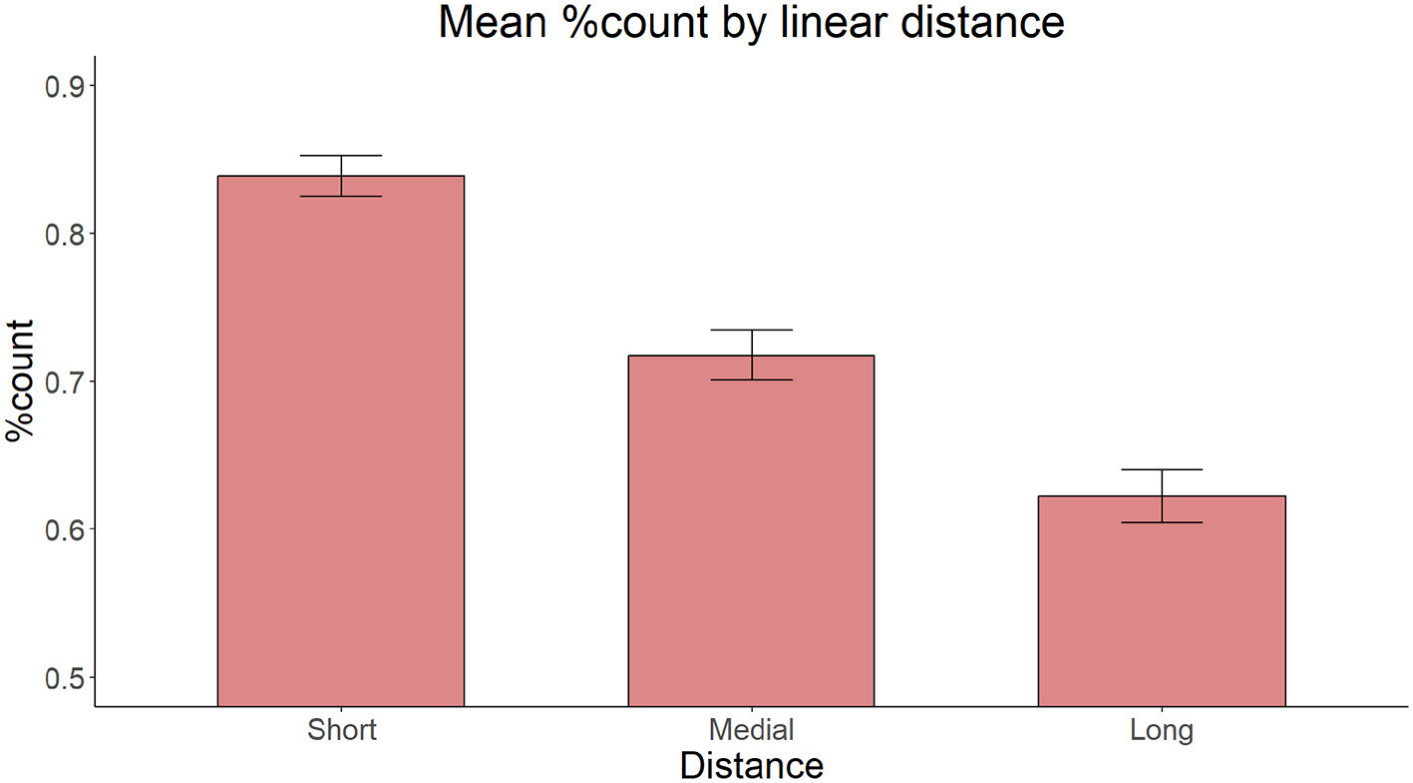
Mean proportion of grammatical count forms produced according to linear distance.

The model with Linear Distance as the only fixed effect yielded a significant main effect of Distance [$\chi_{\left(2\right)}^{2}$ = 129.98, *p* < 0.001]. Pairwise comparisons with the Tukey correction revealed that participants produced fewer count forms in the Medial condition (z = 6.647, *p* < 0.001) and the Long condition (z = 10.940, *p* < 0.001) conditions than in the Short condition, and fewer count forms in the Long Condition than in the Medial condition (z = 4.832, *p* < 0.001). Figure 2 illustrates the proportion of grammatical count form productions in each Distance condition.

The exploratory model with Distance and Gender of the intervening noun yielded a marginal, non-significant Distance x Gender interaction [$\chi_{\left(2\right)}^{2}$ = 4.779, *p* = 0.092, see Appendix A and Section 9.2], with a trend toward fewer count forms in the Long than in the Medial modification condition with feminine intervening nouns, but not with neuter intervening nouns. Exploratory correlations revealed that the more count forms participants produced overall, the less sensitive they were to the presence of an intervening noun [r_(92)_ = −0.53, *p* < 0.001] and to the linear distance [r_(92)_ = −0.26, *p* = 0.013].

### 6.3 Discussion

**Figure 3:**
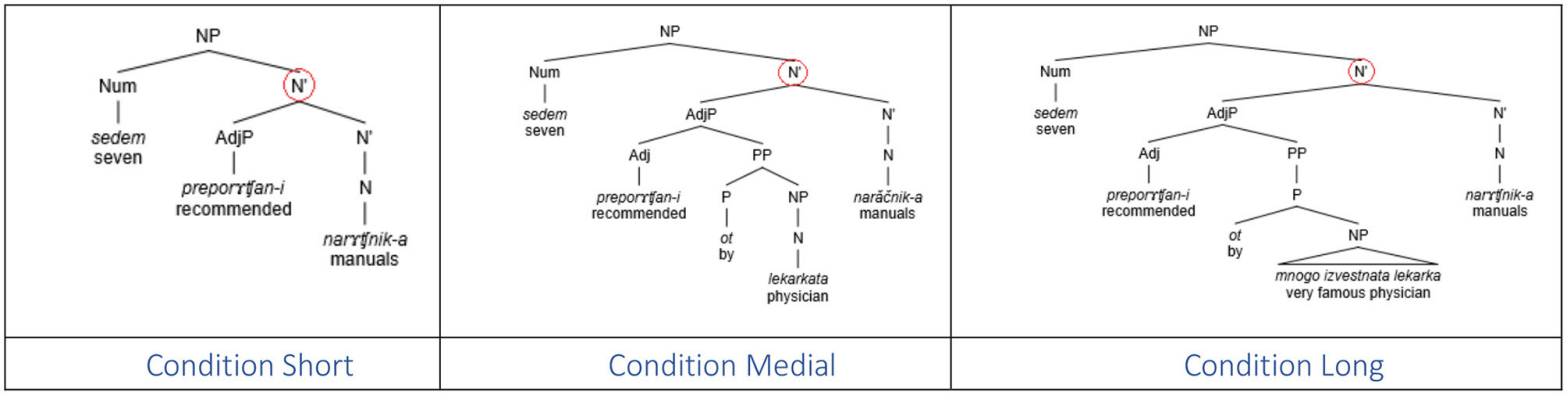
Syntactic structures of sentence-final numeral phrases in Experiment 1.

Experiment 1 revealed a robust linear distance effect in the production of erroneous plural productions in the context of [+count] feature assignment in the Bulgarian numeral phrase. Note that the plural marker in the intervening region occurs only on the first adjective and it is uniform in all three conditions, so the results are not due to cumulative activation of plural marking in the intervening region (see Section 3). Given that the syntactic intervention is also uniform across all conditions, namely, one intervening syntactic node (cf. Figure 3), this suggests that the linear effect is due to the sheer number of intervening lexical items, regardless of their categorical status. We interpret the lack of a significant influence of the gender of the intervening noun as an indication that the similarity of the category of the intervenor and the target of assignment does not modulate the error rate, in contrast to agreement where the same categorical status (noun) of the attractor/intervenor status and the agreement controller was a prerequisite for the attraction effect. We postpone the comments regarding the Gender effect until General discussion (Section 10).

## 7 Experiment 2

In contrast to Experiment 1, which explored the effect of linear distance, the aim of Experiment 2 was to determine whether the production rate of erroneous plural forms in a syntactic context which required a count form is affected by structural distance, similarly to agreement attraction errors. Specifically, we tested the hypothesis that the production of a count form is affected by the number of c-commanding syntactic nodes intervening between the feature assigner (the numeral) and the target noun, where linear distance is kept constant. For clarity, we refer to this hypothesis as the Structural Distance Hypothesis. If the Structural Distance Hypothesis holds in the case of feature assignment, we expect the rate of erroneous selection of the plural form to increase with a greater number of syntactic nodes intervening between the numeral and the noun.

### 7.1 Method

#### 7.1.1 Participants

One hundred and six native Bulgarian speakers (92 females and 14 males, mean age = 45.08, SD = 12.94) who did not take part in Experiment 1, participated in this experiment. They received no material compensation for their participation.

#### 7.1.2 Materials

**Figure 4:**
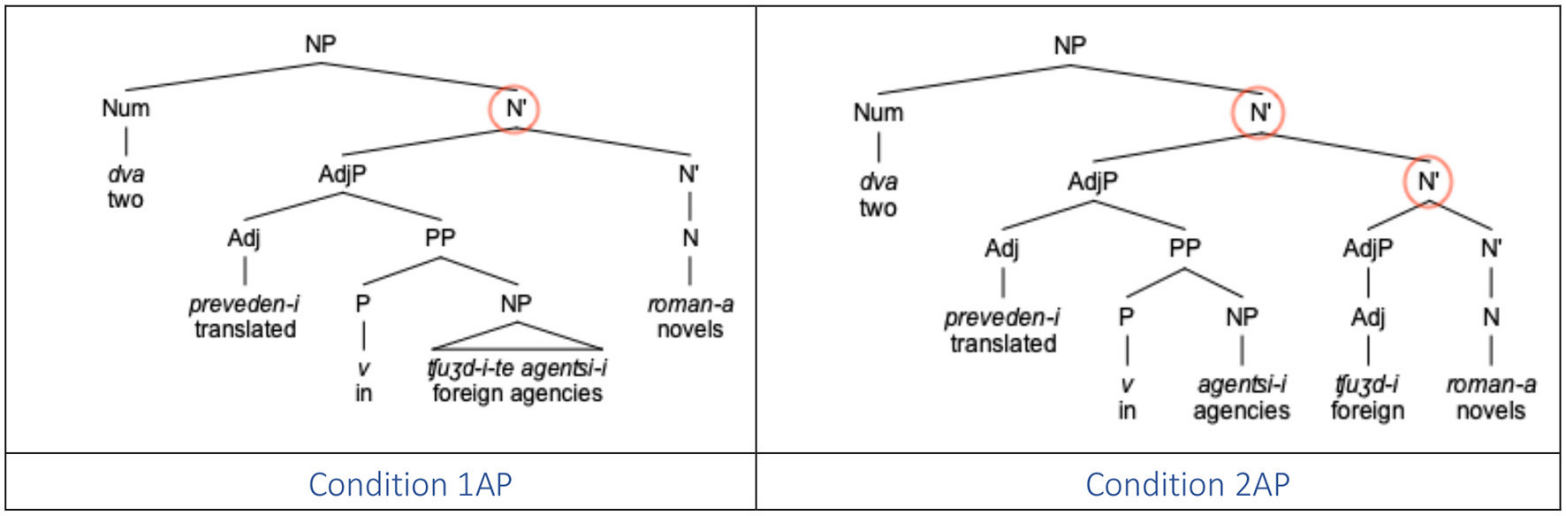
Syntactic structure of a sample numeral phrase in conditions 1AP **(left)** and 2AP **(right)**.

The structure of the materials was similar to that in Barkalova et al. (2018). The materials consisted of 24 experimental items, each made up of a sentential preamble with a sentence-final subject in the form of a numeral phrase. The sentence-final numeral phrase included a numeral, the intervening modifying material, and was missing the final head noun. The missing target noun was the head of the grammatical object of the sentence. The lemma for the final head noun was provided separately on the screen. The intervening modifying material contained one or two adjectival phrases and was constructed in such a manner that one of the adjectives was either modifying a (non-target) noun within the scope of another adjectival phrase (condition 1AP), or constituted its own adjectival phrase thus increasing the structural distance between the numeral and the final noun (condition 2AP). The second intervening plural adjective c-commanded the target noun in the 2AP condition, but not in the 1AP condition. The lexicalization across the two conditions was kept constant, as was the overall number of words in the intervening region of the dependency. All intervening elements were always in the plural form (adjective by virtue of the grammar, nouns by deliberate choice). Whereas all final target nouns were masculine, the gender of the intervening nouns was balanced in such a way that half the intervening nouns were feminine and the other half neuter. An example of an experimental item in the two experimental conditions, corresponding to the interpretations in (10), is provided in Table 2, with syntactic structures in Figure 4.

(10) **Sentence item translations by condition in Experiment 2**
  1AP: The publishing house published two novels translated in the foreign agencies
  2AP: The publishing house published two foreign novels translated in the agencies

**Table 2:** Example of a sentence item in Experiment 2.

**Preamble**	Izdatelstv-o-to publikuv-a publishing.house;N-N;SG-N;SG;DEF publish;PFV-AOR;3;SG;PST	
Condition	Sentence-final NumP, modifier part	Target noun lemma
1AP	dv-a [preveden-i v [ 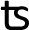 u 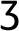 d-i-te] agen 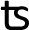 i-i] two;M translated-PL in foreign-PL-DEF;PL agency;F-PL	roman - novel;M;SG (baseform)
2AP	dv-a [preveden-i v agen 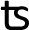 i-i-te] [ 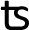 uγd-i] two;M translated-PL in agency;F-PL-DEF;PL foreign-PL	

The grammatically correct form of the target noun in the experimental materials was always count. In order to hide this regularity, the materials also contained 54 filler items with a similar structure to experimental items, all of which required a form that is either plural or morphologically identical to the plural form. These included 18 filler items whose target noun was feminine and modified by a numeral (the count form of feminine and neutral nouns, which is used in the cases when those nouns are modified by a numeral, is morphologically identical to the plural form), 18 filler items whose final noun was masculine and not modified by a numeral, and 18 filler items whose target noun was feminine and not modified by a numeral. This ensured that half of the items (experimental or filler) had a numeral, and that half of the target nouns were masculine while the other half feminine. The numerals in the experimental and filler items were between two and *seven*, each one appearing an equal number of times.

The audio versions of the experimental stimuli were pre-recorded in their entirety following the same procedure as in Experiment 1.

#### 7.1.3 Procedure

The experimental procedure was identical to that of Experiment 1.

#### 7.1.4 Data coding and analysis

Participants' recordings were coded manually by a native speaker of Bulgarian (the first author). Accuracy was analyzed using Generalized Linear Mixed Effects Regression Models with the *lme4* package (Bates et al., 2015) in R (R Core Team, 2018). Main effects and interactions are reported from model comparisons where the minimal difference between two models is the target effect, while pairwise comparisons were obtained using the *emmeans* package.

All models contained Participant and Item as random effects. For all experiments, we report an initial hypothesis testing model containing only the target independent variable(s) as fixed effect(s). These analyses were then followed up with an exploratory model that included the Gender of the intervening noun as an additional fixed effect. Further exploratory analyses consisted of Spearman correlations between overall accuracy and sensitivity to Intervention calculated as ACC_absent_-ACC_present_, as well as sensitivity to linear distance calculated as ACC_Short_-ACC_Long_.

### 7.2 Results

No items were removed from analysis as none showed performance that greatly differed from the overall distribution. Thirty-eight participants produced fewer count forms than plural forms overall (and therefore had < 50% correct responses), but as this was the goal of the experimental manipulation, these participants were not removed. Seven participants produced no count forms at all and were removed from analysis.

**Figure 5:**
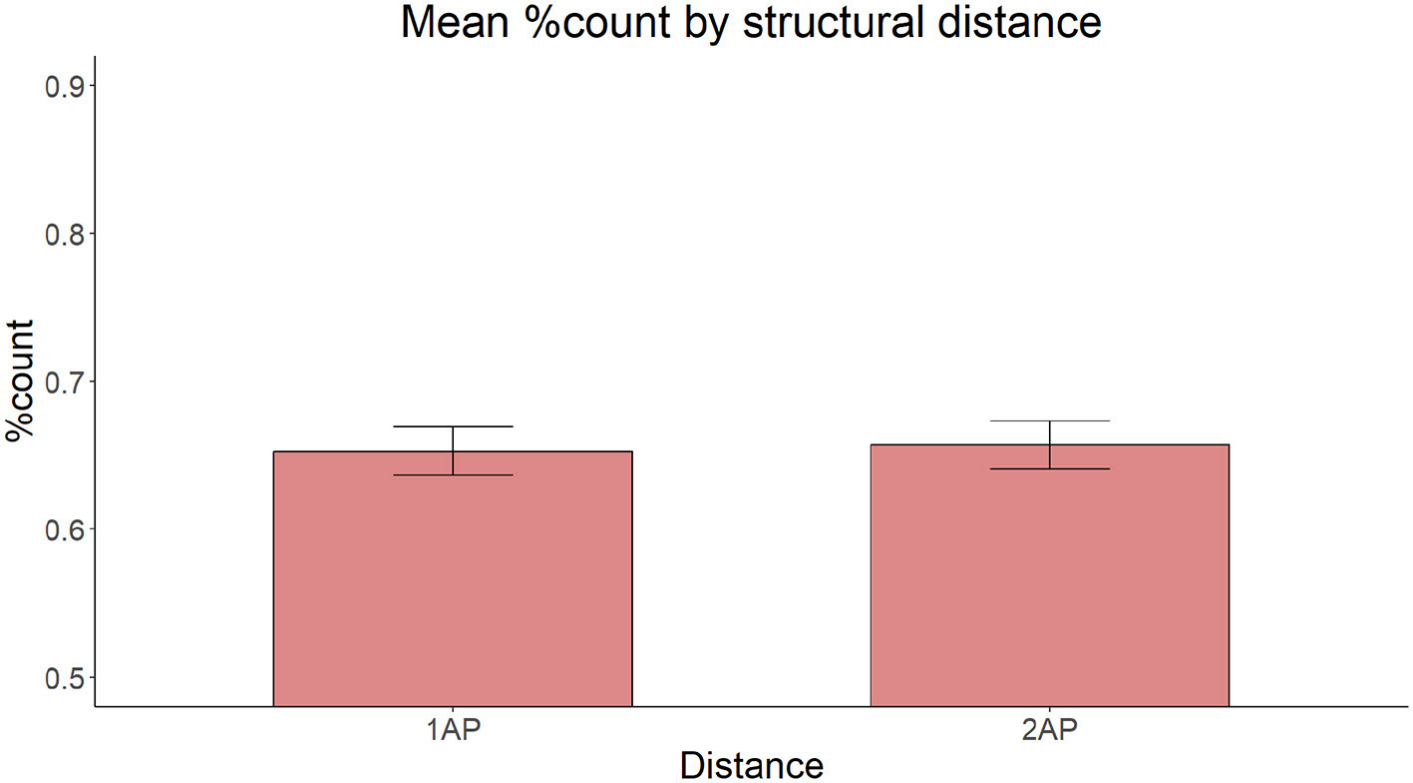
Mean proportion of count forms produced by structural distance.

The model with Structural Distance as the only fixed effect yielded no significant main effect of Distance [$\chi_{\left(1\right)}^{2}$ = 0.0865, *p* = 0.769]. Figure 5 shows the proportion of count forms produced in the Single adjectival and Double adjectival conditions, respectively.

The exploratory model with Structural Distance and Gender of the intervening noun as fixed effects yielded a non-significant trend for a main effect of Gender [$\chi_{\left(1\right)}^{2}$ = 2.300, *p* = 0.129], with more plural productions with neuter than with feminine intervening nouns. Further exploratory correlations revealed no relationship between the overall proportion of count forms produced and the sensitivity to structural distance.

### 7.3 Discussion

The results of Experiment 2 revealed that structural distance (a variable which measures the effect of c-command) does not affect the ratio of production errors in the Bulgarian numeral phrase, which contrasts with the significant effect of linear distance found in Experiment 1. Thus the results showed no effect of structural distance/c-command in case of feature assignment in the Bulgarian numeral phrase. If errors are a marker of processing difficulty, then the structural distance in terms of two intervening syntactic nodes did not contribute to that difficulty. We also note that the overall ratio of assignment errors in both conditions was high (about 35%), similar to that found in the Long condition of Experiment 1 (cf. Figure 3), which featured a single intervening syntactic node and a comparable number of lexical items (3 in Experiment 2, 4 in Experiment 1, see also General Discussion). This again points to the role of linear distance when making the decision regarding the morphological form of the final noun in the time course of processing the feature assigning dependency. Given that the literature in agreement shows that the C-command factor can be used to predict the rate of attraction errors, in particular, in object agreement constructions (Franck et al., 2006), it is relevant to further test the impact of this factor in the Bulgarian numeral phrase. Thus, a second experiment (Experiment 3) focusing on structural distance/c-command was proposed which had a slightly different design I comparison to Experiment 2.

## 8 Experiment 3

Even though in Experiment 2 the linear distance between the numeral and the target noun was controlled for, the linear proximity between the manipulated plural adjective and the target was not (e.g., *tfud-i* was always adjacent to the final noun position and farthest from the numeral in condition 2AP but was distanced further from it and closest to the numeral in condition 1AP, see Table 2). This could have pushed the ratio of errors in either condition in the direction that contributed to an eventual leveling of the error rate in both conditions (the proximity to the numeral might have increased the production errors in the 1AP condition, given that the manipulated adjective itself bears a plural form). In order to address this potential confound and further explore the role of syntactic structure, we ran Experiment 3 which, apart from controlling for the structural distance/c-command metric, also controlled for the proximity metric. Specifically, the position of the target adjective was manipulated across two dimensions: c-command and proximity to the numeral as well as the final noun, to examine whether these factors affect the rate of erroneous plural productions in the numeral phrase.

In addition, Experiment 1 has demonstrated that the linear distance effect could not be affected by the cumulative presence of the plural markers in the intervening region. In Experiment 3 we also explored the possibility that the presence of the *count form* in the intervening region may negatively affect the ratio of plural errors on the final noun, in effect improving the accuracy of the feature assignment process. To that effect, the materials in Experiment 3 also involved manipulation of the morphological marking of the intermediate noun that was presented either in the plural form or in the count form.

### 8.1 Method

In Experiment 3 the following additional factors were manipulated: (i) proximity (a linear position) of the manipulated adjective relative to the final noun (Proximal or Non-Proximal); (ii) the structural position of the manipulated adjective (C-command or No-c-command), (iii) intervening count noun (present or absent). Unlike Experiments 1 and 2, Experiment 3 involved an auto-paced (RSVP) reading task with a two-alternative forced-choice answer (Staub, 2010). In this experiment, both the accuracy of response and response times were measured and used as dependent variables in the analyses.

If the assignment of the [+count] form is sensitive to structural distance similarly to attraction errors, we expected the rate of erroneous selection of the plural form to increase in the conditions with greater structural distance, i.e., we expected more errors in the conditions where the target adjective c-commands the target noun than in the respective conditions where the target adjective does not c-command the target noun. Also, if feature assignment is sensitive to linear proximity we expected more erroneous selection of the plural form in the conditions where the target adjective was proximal to the target noun than in the conditions where it was not. In addition, in the context of a morphological activation account, we expected that an intervening noun in the count form can potentially facilitate the decision for a correct count form on the final noun thus reducing the rate of erroneous plural selection, compared to the conditions in which the intervening noun is in the plural form.

#### 8.1.1 Participants

A total of 101 native Bulgarian speakers (73 females and 28 males, mean age = 39.21, SD = 10.49) who did not take part of the previous experiments or had taken one within the time period of 6 months or more, participated in the experiment, for no material compensation.

#### 8.1.2 Materials

The materials included 36 experimental items, with a sentence-final object in the form of a numeral phrase. The sentence-final numeral phrase in the preamble featured a numeral and intervening material including the adjective whose position was manipulated. Unfortunately, phrase structural restrictions pertaining to the Bulgarian numeral phrase precluded crossing of structural relation, linear proximity and count form as independent factors in a full 2 × 3 × 2 design, so only stimuli in 6 conditions could be constructed. Half of the items contained an intervening (non-target) noun in the plural form (Conditions 1–3) and the other half in the count form (Conditions 4–6). The forms of the final head noun were provided separately on the screen. Examples of the experimental conditions are illustrated in Table 3, for the corresponding interpretations in (11).

(11) **Sentence item translations by condition in Experiment 3**
  1. “The land owners fertilized two low chestnuts planted between the sycamores.”
  2. “The land owners fertilized two chestnuts planted between the low sycamores.”
  3. “The land owners fertilized two low chestnuts planted between the sycamores.”
  4. “The land owners fertilized two low chestnuts planted between several sycamores.”
  5. “The land owners fertilized two chestnuts planted between several low sycamores.”
  6. “The land owners fertilized two low chestnuts planted between several sycamores.”

**Table 3:** Example of a sentence item in Experiment 3.

**Preamble**				Stopan-i-te nator-i-ha the.land.owner;M.PL;DEF fertilize-PFV;AOR-3;PL;PST	
Condition				Sentence-final NumP, modifier part	Target noun lemma
No.	Intervening count quantifier	Proximity of target adjective to target noun	c-command between target adjective and target noun		
1	No	No	Yes	dv-a [nisk-i] [posaden-i me 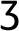 du javor-i-te] two;M low-PL planted-PL between sycamore;M-PL.DEF	kesten- chestnut;M;SG (baseform)
2	No	No	No	dv-a [posaden-i me 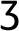 du [nisk-i-te] javor-i] two;M planted-PL between low-PL-DEF;PL sycamore;M-PL	
3	No	Yes	Yes	dv-a [posaden-i me 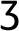 du javor-i-te] [nisk-i] two;M planted-PL between sycamore;M.PL.DEF low-PL	
4	Yes	No	Yes	dv-a [nisk-i] [posaden-i me 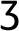 du njakolko javor-a] two;M low-PL planted-PL between several sycamore;M. COUNT	
5	Yes	No	No	dv-a [posaden-i me 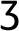 du njakolko [nisk-i] javor-a] two;M planted-PL between several low-PL sycamore;M.COUNT	
6	Yes	Yes	Yes	dv-a [posaden-i me 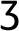 du njakolko javor-a] [nisk-i] two;M planted-PL between several sycamore;M.COUNT low-PL	

The internal structure of the conditions was very similar to that in Barkalova et al. (2018) and Experiment 2. Condition 1 involved two intervening adjectival phrases. In this condition, the manipulated adjective c-commanded the final noun (since it formed the first intervening adjectival phrase) and it was not proximal to the target noun. In contrast, in Condition 2 the manipulated adjective did not c-command the final noun and as it was embedded in the (only) intervening adjectival phrase and it was not proximal to the target noun. Condition 3 was similar to Condition 1 except that the manipulated adjective was proximal to the final noun. Conditions 4–6 were identical to Conditions 1–3 except that they involved a (non-final) noun phrase in the count form, rather than plural (depending on the gender of the noun). The count form was licensed by a non-numeral quantifier such as *njakolko* “several.”

It is important to note that the structural and linear distance between the manipulated adjective and the target noun, as well as the structural distance between the numeral and the target noun varied across conditions, whereas linear distance between the numeral and the target noun is kept constant across conditions with and without an intervening count quantifier. Intervening elements other than the noun phrase modified by the count quantifier (participle/adjective, noun) were always in the plural, as before. All phrase-final nouns were masculine. A third of the intervening nouns were masculine (and thus appearing in a count form in the conditions with an intervening count quantifier), a third were feminine and the last third were neuter (these were always in form that is morphologically identical to the plural form, even in the condition with an intervening count quantifier, since the count form of feminine and neutral nouns is morphologically identical to the plural form).

The grammatically correct form of the target noun in the experimental materials was always count. In order to hide this regularity, the experiment contained 72 filler items which required either a plural or a count masculine noun. The fillers were factored by the presence of the numeral as well as complexity. Unlike the experimental items, each filler was constructed in only one of the six versions mirroring the experimental condition. Each item in one group of fillers had a very similar structure to the experimental items except there was no initial numeral modifying the final noun, so that the latter appears in plural form. There were 54 filler sentences with no numeral and the correct plural form, and 18 sentences involving the numeral and correct count form on the noun, so the overall number of experimental and filler items was therefore balanced equally across the two marking possibilities for the final noun (count or plural), in order to avoid a potential bias. The experimental and filler items were also balanced by the gender of the noun in the intervening region (masculine, feminine, or neuter). A different group of fillers had a simpler structure than that of experimental items by not including a second (proximity-manipulated) adjective; there was only one adjectival phrase modifying the final noun. A more detailed overview of the filler scheme for Experiment 3 can be found in Appendix B. The numerals used in the experimental and filler items were between two and seven, each appearing an equal number of times.

#### 8.1.3 Procedure

The experiment was implemented in the Ibex Farm platform. Participants had to press a button to launch the presentation of a preamble which was displayed on the screen word by word with the words appearing in the middle of the screen. Each word was displayed for 650 ms with no inter-stimulus interval. Immediately after the last word of the preamble was shown, two different forms of the final missing noun appeared on the screen. One option was the count form of the target noun and the other was its plural form. The order of the count and the plural forms on the screen (left or right) was randomized. Participants were asked to press a response button to choose the correct form of the final noun.

The items were presented in a randomized order. Each participant saw every experimental and filler item once, in one of the six conditions; conditions were balanced across lists.

#### 8.1.4 Data analysis

Generalized linear mixed effects regression models were built in the same way as in the previous experiments, always containing Subject and Item as random effects. In Experiment 3, the fixed effects of the initial morel were Proximity of the target adjective and the target noun (proximal vs. non-proximal), Relationship between the target adjective and the target noun (c-command vs. non-c-command) and Presence or absence of a numeral for the target adjective (present vs. absent).

Reaction times for Experiment 3 were analyzed with Linear Mixed Effects Regression Models in R (R Core Team, 2018), using the lme4 package (Bates et al., 2015). These models contained Subject and Item as random effects, and Proximity of the target adjective and the target noun (proximal vs. non-proximal), Relationship between the target adjective and the target noun (c-command vs. non-c-command) and Presence or absence of a numeral for the target adjective (present vs. absent) as fixed effects. Main effects and interactions we**r**e obtained from *anova* (model) function, while pairwise comparisons were realized using the *emmeans* package.

### 8.2 Results

Two hundred and seventy (7.42%) observations were removed due to extremely slow (>7,500 ms) reaction times. No items were removed from analysis as none showed performance that greatly differed from the overall distribution. Four participants chose fewer count forms than plural forms overall (and therefore had < 50% correct responses), but as this was the goal of the experimental manipulation, these participants were not removed.

**Figure 6:**
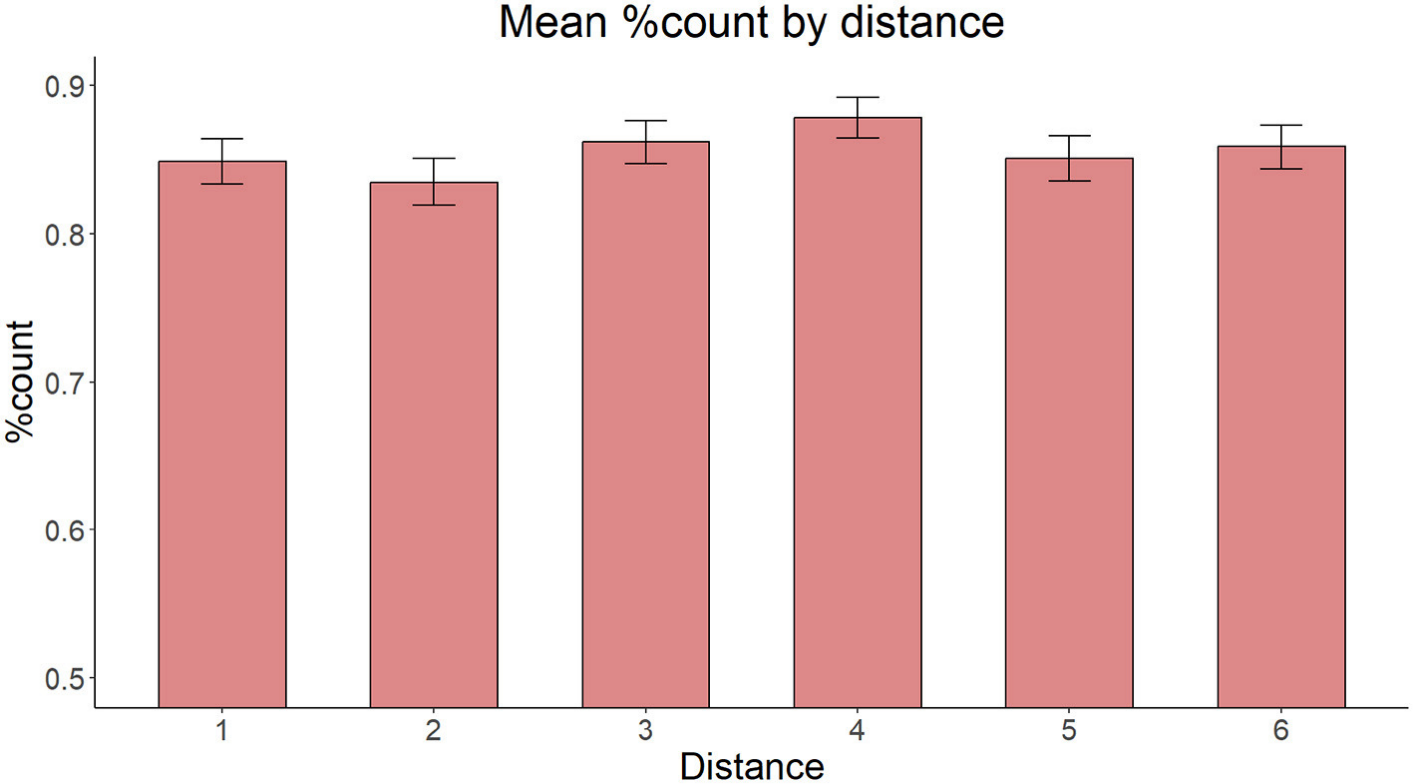
Mean proportion of count forms produced by Proximity of the target adjective and the target noun.

**Figure 7:**
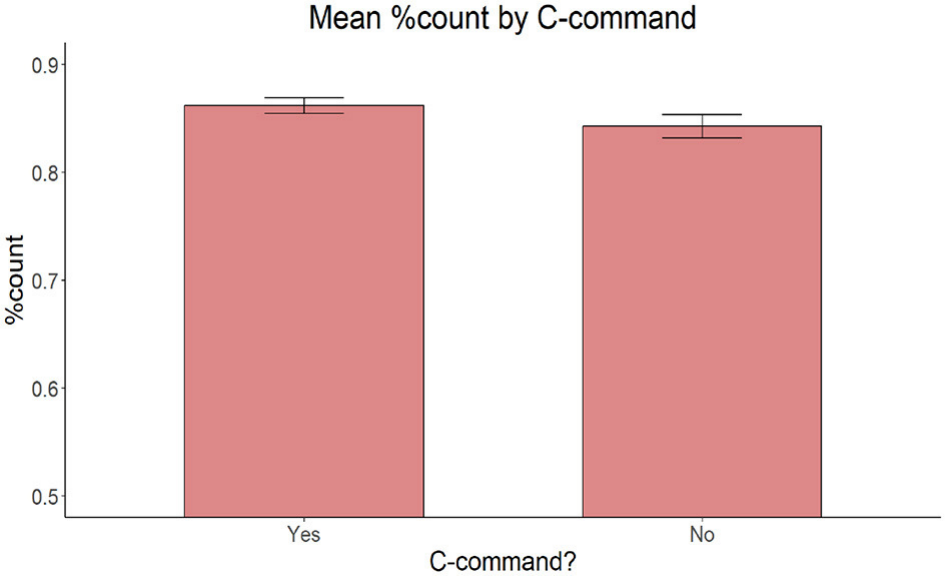
Mean proportion of count forms produced by C-command relationship between the target adjective and the target noun.

**Figure 8:**
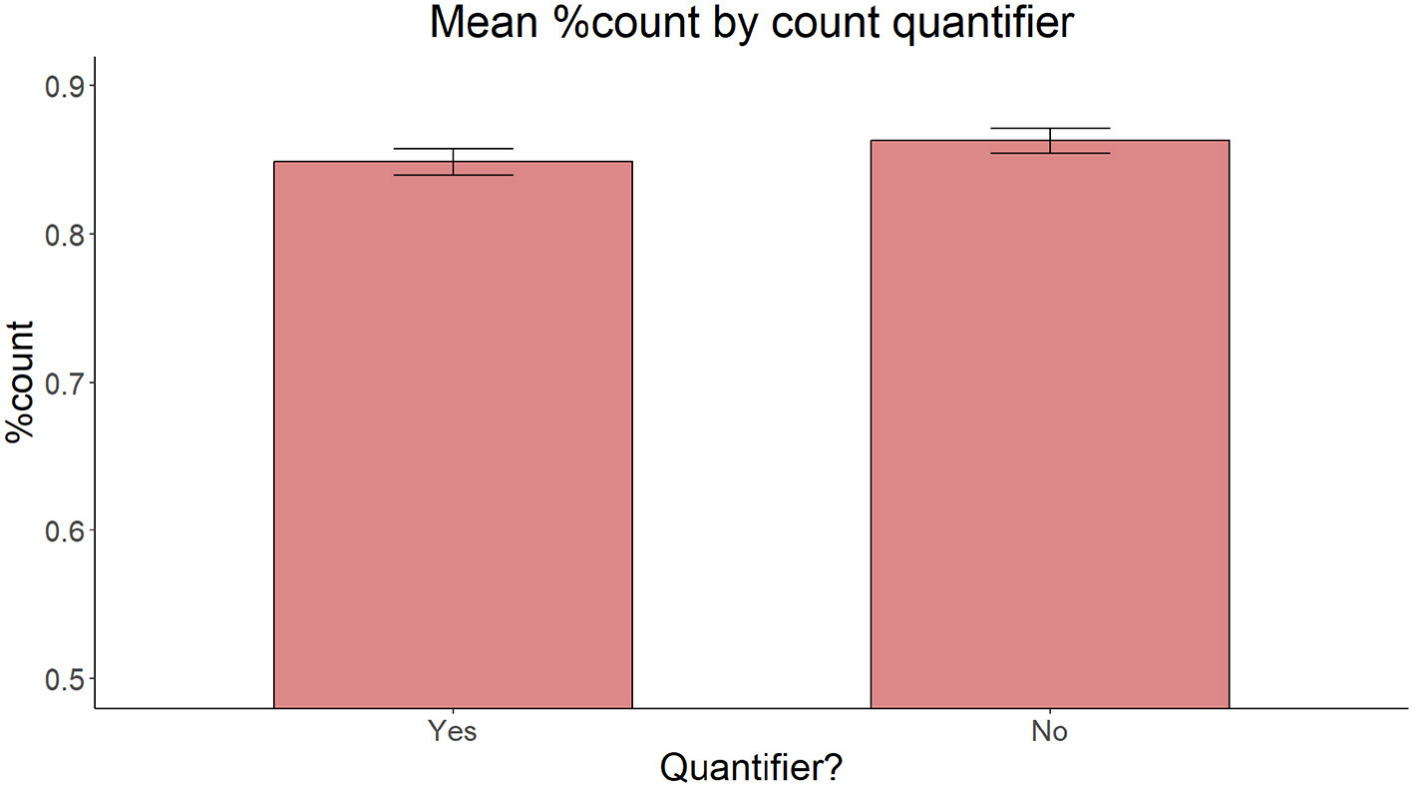
Mean proportion of count forms produced by presence or absence of an intervening count quantifier.

On accuracy, the model with Proximity of the target adjective and the target noun (proximal vs. non-proximal), Relationship between the target adjective and the target noun (c-command vs. non-c-command) and Presence of an intervening count quantifier (present vs. absent) as fixed effects yielded no significant main effect of Proximity [$\chi_{\left(2\right)}^{2}$ = 2.696, *p* = 0.260], C-command [$\chi_{\left(2\right)}^{2}$ = 0.350, *p* = 0.554], or Intervening count quantifier [$\chi_{\left(1\right)}^{2}$ = 1.6221, *p* = 0.203] (Figures 6–8).

**Figure 9:**
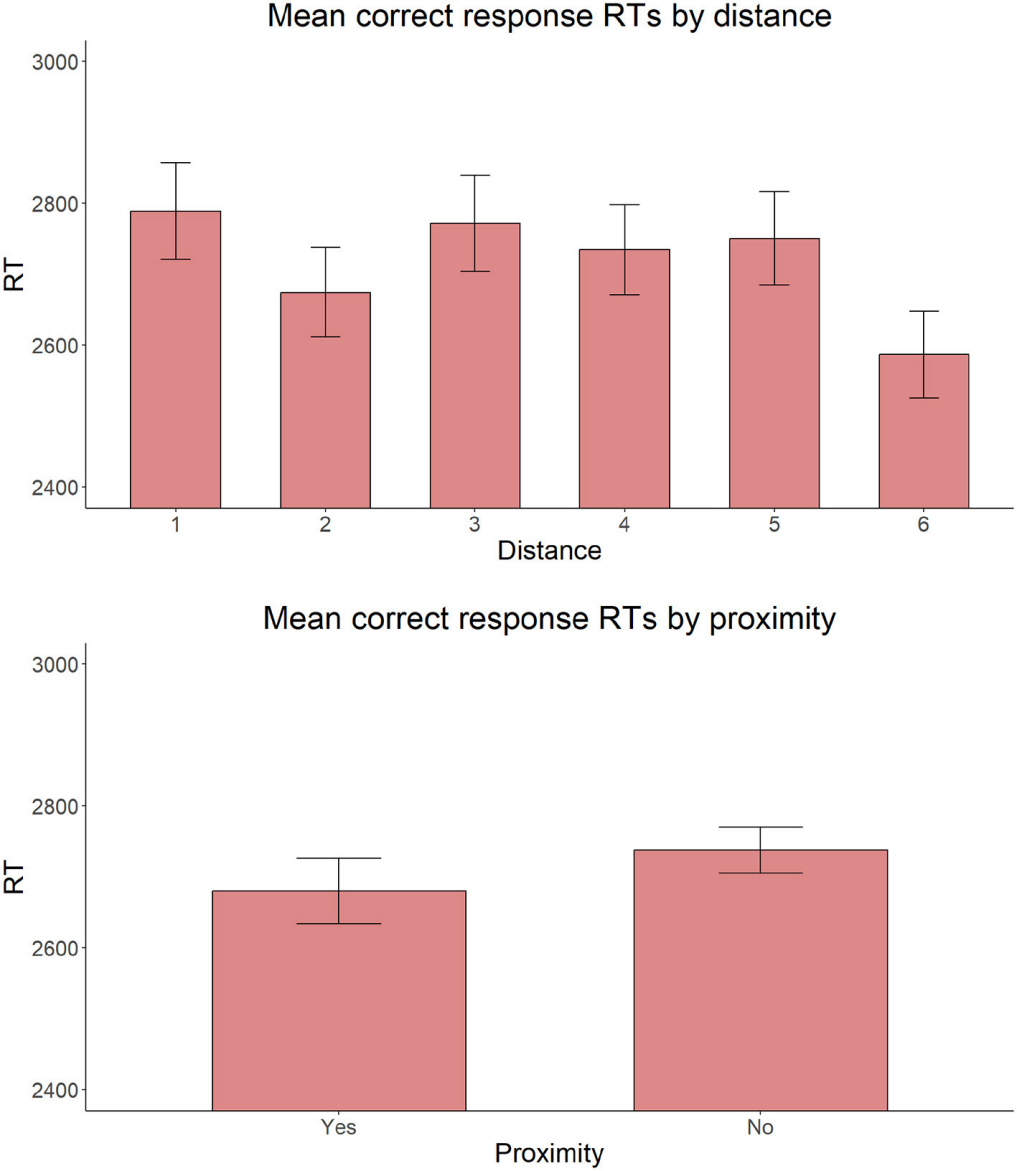
Mean RTs for correct response trials by distance and proximity of the target adjective and the target noun.

**Figure 10:**
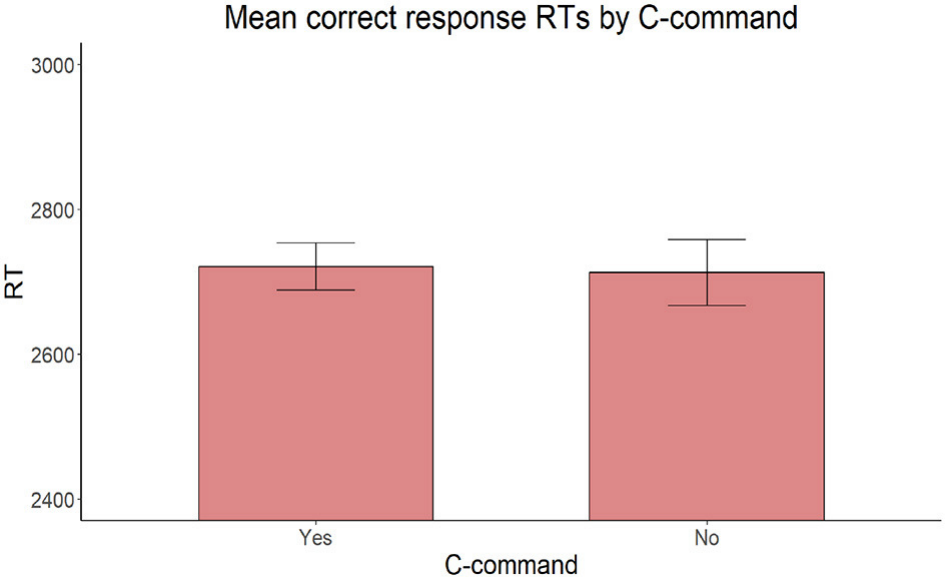
Mean RTs for correct response trials by C-command relationship between the target adjective and the target noun.

**Figure 11:**
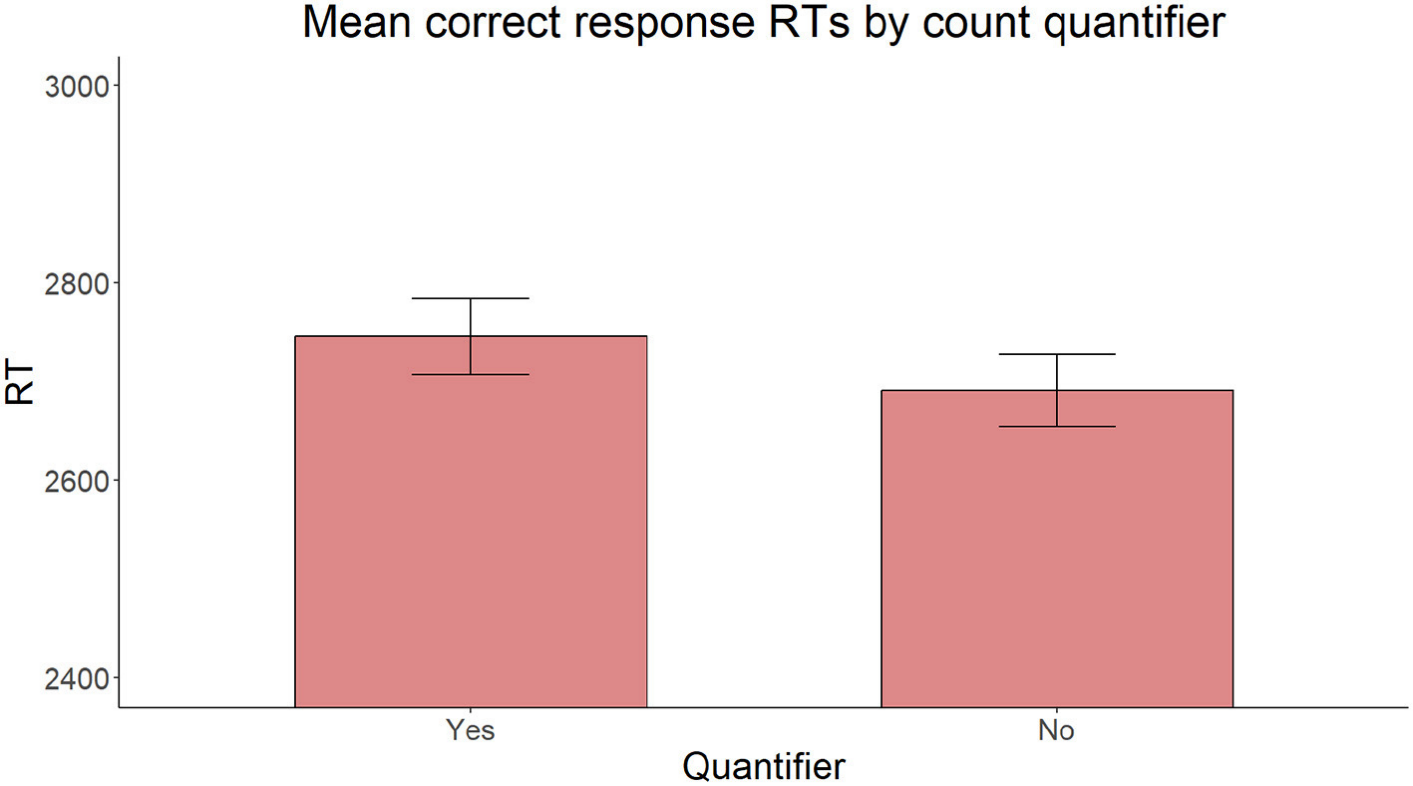
Mean RTs for correct response trials by presence or absence of an intervening count quantifier.

Also, on reaction times for correct responses, the model with the same fixed factors yielded no significant main effect of Proximity [$\chi_{\left(2\right)}^{2}$ = 2.306, *p* = 0.129], C-command [$\chi_{\left(1\right)}^{2}$ = 0.127, *p* = 0.722], or Intervening count quantifier [$\chi_{\left(1\right)}^{2}$ = 2.002, *p* = 0.157] (Figures 9–11).

### 8.3 Discussion

The results of Experiment 3 showed a remarkable uniformity in the context of variability imposed by manipulation of structure-related factors. The prediction for more errors in the conditions where the target adjective c-commands the target noun than in the respective conditions where the target adjective does not c-command the target noun was not supported. Thus, Experiment 3, which now had an improved design that controlled for linear proximity, replicated the results of Experiment 2, even when the proximity of the manipulated adjective was controlled for. In addition, an expectation in the spirit of a morphological activation account that the count form on the intervening noun would push the rate of erroneous plural production down was not supported either. From the perspective of agreement attraction literature, these results seem surprising. They suggest that structure-dependence as well as interference in the intervening region have no influence on the feature assignment dependencies in Bulgarian numeral phrases, at least as far as potential improvement of performance due to intervening count form markers is concerned. In this respect, recall also that Experiment 3 employed a forced-choice paradigm, whereas Experiments 1 and 2 elicited spoken production. The converging null interference effect across paradigms strengthens our interpretation, but a future replication of the six-condition design in a production format would provide an even tighter methodological match and remains a natural next step for this line of work.

Experiment 3 used nouns in the plural forms as potential intervenors to the count form assignment. In the next experiment, we further investigate the issue of featural interference in the “opposite” context involving intervening nouns bearing the count form, instead. For this, we turn to the construction known as nominal concord which provides a natural backdrop to the feature assignment contexts that we have been exploring so far.

## 9 Experiment 4

In the previous experiments, we explored whether intervening plural feature(s) affect the assignment of the count form by the numeral under structural and linear intervention configurations. We have seen that linear, but not structural, distance determines the rate of the production errors in the Bulgarian numeral phrase. We have also seen that the count form morphology on an intervening noun does not affect the ratio of production errors in the context of assignment of the count feature by the numeral. In this last experiment, we compare performance in the feature assignment context with that in the closely related agreement context of nominal concord. Recall that in the numeral phrases considered so far, a potential agreement/concord relation was “embedded” in a feature assignment relation [see (8) and Section 3 above]. Here, we want to look at the opposite case of a feature assignment relation embedded in a concord relation. Specifically, we were interested in (i) whether a similar linear distance effect obtains also in the concord relation, and (ii) whether an intervening count form triggers interference in agreement/concord, i.e., attraction errors. A schematic representation of the dependency of interest is in (12).

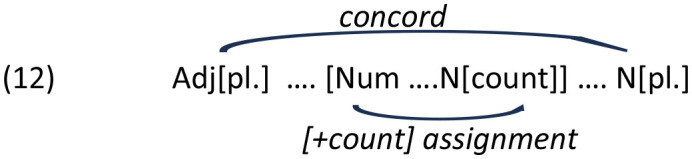

Similarly to English, Bulgarian is a language where adjectives precede their head noun. At the same time, being a morphologically rich language, Bulgarian overtly marks adjective-noun agreement on the adjectives. Also, Bulgarian allows complex adjectival modifiers to intervene between the prenominal adjective and the head noun. Having adjectives agreeing with the head noun, as well as complex adjectival modifiers between the prenominal adjective and the head noun, makes Bulgarian suitable for studying attraction errors in adjective-noun agreement. The aim of this experiment was therefore to explore whether linear distance as well as intervening count form morphology influence potential agreement errors in the concord structures.

In sum, while Experiments 1–3 tested a particular feature assignment configuration within the numeral phrase, Experiment 4 tests, essentially, an agreement configuration. From the agreement attraction literature, we know that agreement is essentially a memory process based on retrieval of the cues of the agreement controller at the agreement target (Section 1). As in subject-verb agreement, nominal concord is likely computed at the point of the noun via re-accessing the relevant features of the adjective on the basis of similarity of respective morpho-lexical cues. In languages like Bulgarian where adjectives precede nouns, the order of controller and target are reversed in the agreement/concord structures, but this may still be in line with the scenario when the agreement cues encoded at the adjective are re-activated when processing the noun, as the results from morphological priming studies also suggest (Section 3). We also know that cue-based retrieval is sensitive to interference from other elements temporarily stored and carrying syntactic features that are similar to those of the target. Thus, if the nominal agreement/concord structure we are testing is driven by similar mechanisms as that in subject-verb agreement, we can expect similar attraction effects, as manifested by a feature mismatch effect.

On the other hand, accounts based on the idea of cumulative activation (cf. Section 3) would predict that intervening plural adjectives whose plural feature matches the plural feature of the initial adjective would actually *facilitate* the production of the plural form on the noun. As the number of intervening adjectives increases, the number of plural forms also increases, and the number of errors is thus expected to decrease due to a cumulative “feature match” or facilitation effect between the initial plural adjective and the plural intervening adjective. This facilitation effect may create a bias toward the use of the correct plural form on the target noun. The two predictions go in the opposite directions but together provide an interesting context for tracing the dynamics of potential involvement of both interference mechanisms and those involved in the cumulative activation/facilitation effects in the Bulgarian nominal concord structure.

### 9.1 Method

#### 9.1.1 Participants

A total of 94 native adult Bulgarian speakers (66 females and 28 males, mean age = 37.83, SD = 12.47) who did not participate in Experiments 1–3 or similar experiments within the period of 6 months or more, participated in the experiment for no material compensation.

#### 9.1.2 Materials

**Figure 12:**
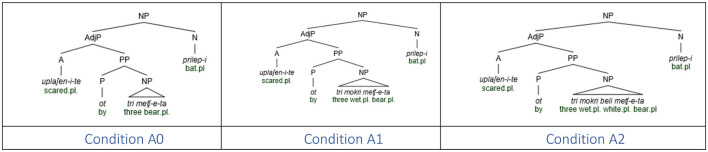
Syntactic structure of sentence-final numeral phrases in Experiment 3.

The materials consisted of 24 experimental items, each made up of a sentential preamble ending with a sentence-final subject in the form of a noun phrase containing a numeral phrase as its component part. The sentence-final noun phrase included an adjective/participle, the intervening modifying material consisting of a prepositional phrase with a full numeral phrase containing a numeral and a noun modified by a number of adjectives varying between zero and two, as the complement of the preposition. The final head noun was missing in the stimuli, as in the previous experiments. All intervening elements (participles/adjectives, noun), as well as the first adjective/participle and the target noun were either plural or in a form that was morphologically identical to plural. Thus, there were three experimental conditions based on the number of adjectives (A0 = none, A1 = one, A2 = two) modifying the noun in the embedded numeral phrase. It is important to note that, as the number of adjectives increases within the numeral phrase, only the linear distance between the numeral and the final noun increases, but not the structural distance, because of the embedded status of the numeral phrase with respect to the final noun. Whereas all target nouns were masculine, half the intervening nouns were feminine and the other half neuter. The fact that the intervening nouns were feminine and neuter ensured that they all had a morphological form that is identical to the plural form (even though they were followed by a numeral) since the count form of feminine and neutral nouns is morphologically identical to the plural form. Examples of experimental items in the three experimental conditions, with the interpretations in (13), is provided in Table 4, Figure 12.

(13) **Sentence item translations by condition in Experiment 4**
A0: In the cave, the crew photographed the bats scared by three bears.
A1: In the cave, the crew photographed the bats scared by three wet bears.
A2: In the cave, the crew photographed the bats scared by three wet white bears.

**Table 4:** Example of a sentence item in Experiment 4.

**Preamble**	V peʃter-a-ta ekip-γt zasn-e… in cave;F.DEF;SG crew;M.DEF.;SG photograph-PFV;AOR;3;SG;PST	
Condition	Sentence-final AP, modifier part	Target noun lemma
A0	uplaʃen-i-te ot tri me 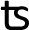 -e-ta scared-PL-PL;DEF by three small.bear;N-N-N;COUNT	prilep - bat;M;SG (baseform)
A1	uplaʃen-i-te ot tri mokr-i me 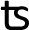 -e-ta scared-PL-PL;DEF by three wet-PL small.bear;N-N-N;COUNT	
A2	uplaʃen-i-te ot tri mokr-i bel-i me 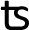 -e-ta scared-PL-PL;DEF by three wet-PL white-PL small.bear;N-N-N;COUNT	

The grammatically correct form of the target noun in experimental materials was always plural. The experiment also contained 72 filler items with a structure similar to that of experimental items, all of which required a target noun in a plural form: 24 filler items whose target noun was feminine and whose intervening noun was modified by a numeral, 24 filler items whose final noun was masculine and whose intervening noun was not modified by a numeral, and 24 filler items whose target noun was feminine and whose intervening noun was not modified by a numeral. This ensured that half of the total number of items (experimental or filler) had a numeral within their nominal intervener and the other half did not. The design also ensured that half of the total number of target nouns were masculine and the other half were feminine. The nominal interveners with a numeral were in a count form that is morphologically identical to the plural form, since the count form of feminine and neutral nouns is morphologically identical to the plural form. The numerals in the experimental items were between two and seven and each type of numeral appeared an equal number of times. Because the grammatically expected response in every critical item was in the plural, we acknowledge that some speakers could in principle show an overall bias toward the plural. Crucially, however, each participant produced the same number of trials in every experimental condition, and random intercepts for participants in our models are likely to absorb this baseline bias. Consequently, any global preference is distributed evenly across conditions (number of intervening adjectives) and can only reduce, not spuriously inflate, the observed condition contrasts.

#### 9.1.3 Procedure

The experimental procedure was identical to that of Experiment 1 and 2. Accuracy of response was the only dependent variable in this experiment.

### 9.2 Results

No items were removed from analysis as none showed performance that greatly differed from the overall distribution. Nine participants produced more count forms than plural forms overall (and therefore had < 50% correct responses). Two participants produced no plural forms at all and were removed from analyses.

**Figure 13:**
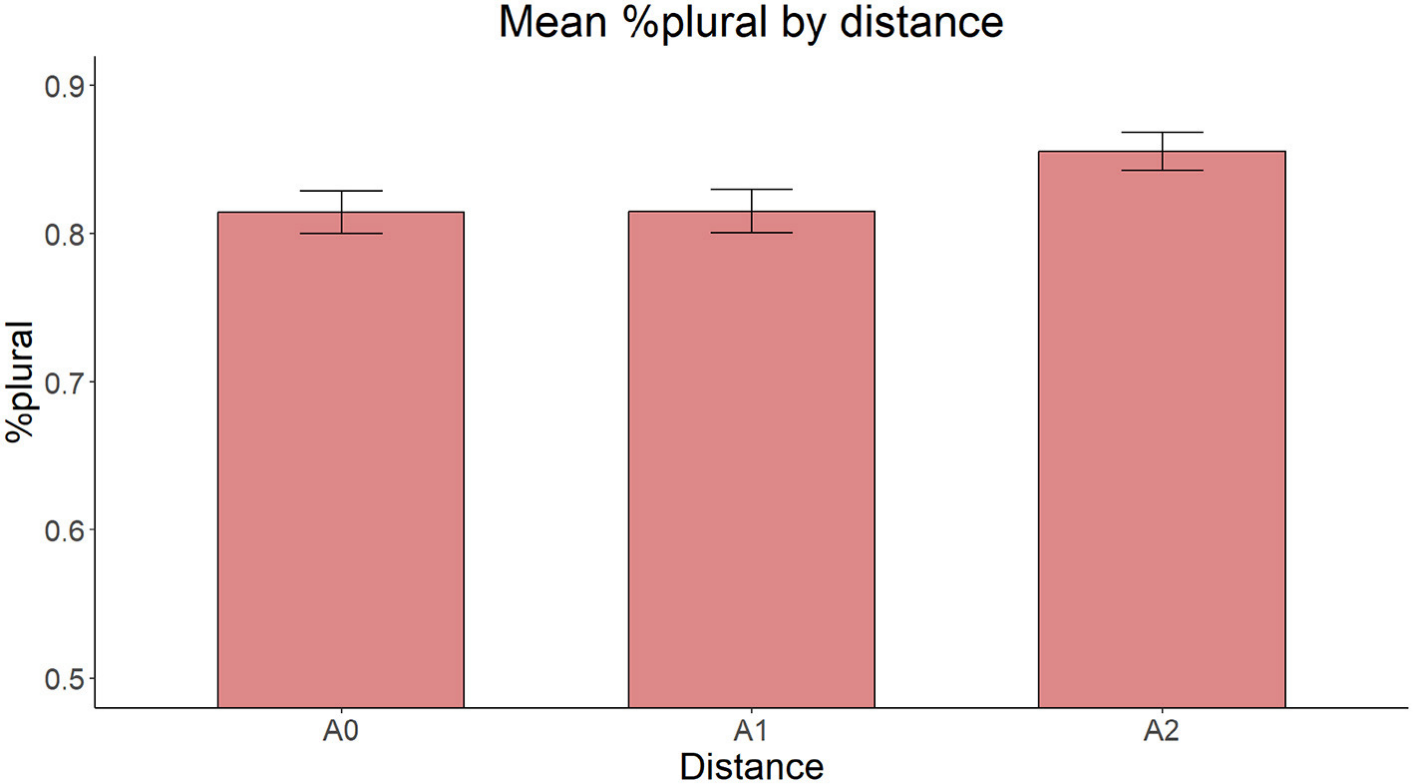
Mean proportion of plural forms produced by distance.

The distribution of the plural accuracy rates for all three conditions is depicted in Figure 13. The model with Distance as the only fixed effect yielded a significant main effect [$\chi_{\left(2\right)}^{2}$ = 8.045, *p* = 0.018]. Pairwise comparisons with the Tukey correction showed that participants produced fewer plural forms in A1 (z = 2.506, *p* = 0.033) and A0 (z = 2.556, *p* = 0.029) conditions than in the A2 condition, with no significant difference between the A0 and A1 conditions (z = 0.046, *p* = 0.999).

The model with Distance and Gender of the intervening noun as fixed effects yielded a significant main effect of Gender showing fewer plural productions for feminine than for neuter interveners [$\chi_{\left(1\right)}^{2}$ = 8.124, *p* = 0.004; Appendix A]. Further exploratory correlations revealed that the more plural forms participants produced overall, i.e., the more accurate they were, the greater their sensitivity to distance: the greater the difference between the A0 and A2 conditions [r_(92)_ = 0.27, *p* = 0.001], the A1 and the A2 conditions [r_(92)_ = 0.23, *p* = 0.031], but not between the A0 and A1 conditions [r_(92)_ = −0.029, *p* = 0.78)].

### 9.3 Discussion

The results of Experiment 4 revealed two important findings. First, as expected, participants do make errors in nominal concord productions when the mismatching count form is intervening on the dependency established by nominal concord. Second, the results showed a clear tendency for facilitating the production of concord in the presence of matching feature(s). In particular, the ratio of error declines in the two adjective (A2) condition compared to the one and no adjective conditions (A1 and A0). There was, however, no difference in accuracy between the A0 and A1 conditions. Given the fact that the two factors, that is, the number of plural markers and the presence of a feature mismatching (count form) intervenor, affect the production in opposite directions, the lack of difference between A0 and A1 can be interpreted to mean either that (i) these factors play no role or (ii) they play a similar role and cancel each other out. There is a reason to think that (ii) is on the right track. The very occurrence of errors in plural agreement (at a rate close to 20% according to Figure 13) does not allow us to dismiss the second factor, and the observed facilitating tendency in the condition A2 reinforces the first one.

What is the role of linear distance in these patterns? In contrast to Experiments 1–3, the results of Experiment 4 suggest the opposite link between correct productions and linear distance: the more accurate participants are, the more they are sensitive to linear distance between the numeral and the noun in the intervening region. The inverse direction of this effect may be due not to linear distance *per se* but to its co-varying factor, i.e., number of plural adjectives, such that more accurate participants would “make better use” of the presence of a plural adjective on the intervener to correctly agree the target noun in the nominal concord.

Experiment 4 also revealed a significantly greater rate of non-plural errors with feminine intervenors than with neuter intervenors. This contrasts with Experiments 1 and 2 that did not show a significant effect of gender in modulating erroneous plural productions (Appendix A). It appears then that the features of an intervening noun actually affect the error rate in the nominal concord constructions, but not in those involving feature assignment.

In sum, the error patterns in the Bulgarian nominal concord construction with an embedded numeral phrase suggest that processing the latter shows the signs of the familiar attraction phenomenon, similar to the one observed in clausal environments. In particular, linear distance does not appear to be a key player in this case, unlike in the feature assignment one, but to (negatively) correlate with the number of plural markers facilitating the resolution of the nominal concord, in line with the activation models. At the same time, an intervening count form affects the ratio of agreement/concord errors. This is similar to the production of subject-verb agreement where match/mismatch effects were reported (Bock and Miller, 1991; Hartsuiker et al., 2003; Vigliocco et al., 1995 among others). This suggests potential key differences in the processing of nominal agreement and of feature assignment dependencies. We elaborate on those in more detail in the next section.

## 10 General discussion

### 10.1 Summary of the results

The results of Experiments 1–3 indicate that production errors in feature assignment in the numeral phrase are sensitive to the linear distance between the feature assigner (numeral) and the assignee (final noun) in terms of number of intervening lexical items which in our case included a noun and modifying adjective(s). At the same time, structural considerations such as the number of intervening syntactic nodes does not seem to affect the feature assignment. This is in opposition with the findings in the literature on subject-verb agreement attraction which acknowledges some degree of linear distance modulating the error ratio but is mostly sensitive to the structural relation such as c-command of the intervener to the target.

In Experiment 4 we investigated production errors in a related construction involving nominal concord. Being an agreement construction, it expectedly manifested a pattern of errors similar to those found in clausal agreement constructions. These errors were modulated by an intervening noun with a mismatching (count) feature. Experiment 4 also revealed the significance of a cumulative activation effect facilitating the choice of the plural ending. At the same time, linear distance does not seem to play a key role in the nominal concord structure.

A further indication that in nominal concord, but not in feature assignment, we are dealing with an interference effect was the gender effect of the intervening noun found in Experiment 4 as opposed to no effect in the other experiments. Slioussar and Malko (2016) found that the gender of the intervening noun affects the rate of attraction errors in Russian clausal agreement constructions. Interestingly, in their production experiment, the feminine gender triggered strongest attraction effects evidenced by most production errors, followed by masculine and neuter interveners (in comprehension, however, the order was different; see the above work for details). The authors interpret this result in terms of feature markedness in the sense that marked features have more chances to be retrieved in agreement (see also Badecker and Kuminiak, 2007). Their result is consistent with the gender effect we found in the nominal concord sentences where more errors were observed with feminine intervenors than with neuter ones suggesting a similar “feminine >> neuter” markedness hierarchy in Bulgarian. In contrast, in Experiment 1 and 2, set in the feature assignment context, there was no conclusive evidence to this effect, although Experiment 1 did show a trend for a similar asymmetry between feminine and neuter intervenors, which, however, did not reach significance. Therefore, the parallel remains tentative for the time being and is subject to further experimentation in order to be strengthened as fully viable evidence.

Thus a side-by-side evaluation of the error patterns in the feature assignment and nominal concord cases across the four experiments indicates that the latter, but not the former, displays the pattern typical of agreement attraction errors. This suggests, in particular, that theoretical accounts of these errors in terms of memory mechanisms, specifically, pertaining to (faulty) retrievals, may potentially explain the interference/attraction effects in the nominal concord structures but not obviously so in the feature assignment dependencies. Feature assignment errors in the context of the Bulgarian numeral phrase seem to be of a different nature. Interference in terms of *features* does not seem to play a significant role here. The lack of such interference effect appears to be related to the properties of the controller: the fact that the controller is a numeral which lacks an overt number feature seems to prevent the number features from the interveners (even if they are overt) to interfere in the attraction process. Thus, no feature mismatch effect is observed in feature assignment. Incidentally, this may also explain the lack of structural effects in feature assignment: if there is no “offending” or mismatching feature in the intervening material, then a particular syntactic configuration of the latter in relation to the final noun is no longer relevant, as far as erroneous production is concerned (see also below).

### 10.2 Feature assignment as morphological prediction

Why is there no interference/attraction effect in feature assignment? Answering this question requires a clearer understanding of feature assignment as a process in real time. Our findings in this study suggest the following potential scenario. Similarly to clausal-level agreement, we take feature assignment to be a process managed in the memory. The difference between the two may stem from what kind of encoded object is actually re-activated at the retrieval stage when completing the dependency. When processing the target of agreement (e.g., verb), the agreement controller's feature(s) are re-accessed in the memory as part of the encoded controller itself on the basis of cues overtly manifested in the input. In contrast, in feature assignment, the [+count] feature, which is part of a morphosyntactic assignment rule of the numeral, is encoded as *a morphological prediction* of the numeral. This prediction can be formalized in the lexical entry for the corresponding numeral in the form of an abstract morphological specification for the [+count] morphology. The following is a possible (partial) feature structure for the numeral *tri “*three” in Bulgarian:

(10) *tri*:NUM
_N [+count]

In the component responsible for morphological encoding, the [+count] specification is realized as a prediction for a particular morphological exponent on a masculine noun or one that is homophonous with the plural one on a feminine or neuter noun. It is this abstract featural prediction that must be completed by supplying the noun in the appropriate form. Thus the processing distinction between agreement and assignment may lie in the nature of the object to be encoded and later retrieved: in the agreement case, it is (the relevant portions of) the lexical item, part of the input, while in the feature assignment case, it is a piece of grammatical knowledge activated by the processing system when encoding the feature licensor. This aspect of encoding can be seen therefore as a “bottom-up” step in the case of agreement whereas in the case of feature assignment it is, rather, “top-down,” in line with its predictive character (cf. Ferreira and Qiu, 2021).

The conjecture that the overt feature from the intervener cannot be transferred whenever the feature assigner lacks an overt number feature accounts for the lack of cue-based interference from any lexical item in the intervening region between the feature assigner and the final noun. It also accounts for the lack of any sort of feature “transmission” or propagation (either from the intervener to the verb or from the intervener to the subject) assumed in many accounts of agreement attraction such as Feature Percolation (Franck et al., 2002; Vigliocco and Nicol, 1998), Marking and Morphing (Eberhard et al., 2005), Feature Controller and Selection model (Franck, 2017), or Self-Organized Sentence Processing (Tabor and Hutchins, 2004; Smith et al., 2018).

Hypothetically, in a numeral phrase containing any number of adjectival modifiers between the numeral and the noun, the predicted abstract [+count] specification may need to “wait” to be realized on the noun indefinitely long. The linear distance effect modulating the ratio of errors observed in our experiments is likely to reflect the processing cost of storing and maintaining the encoded abstract morphological prediction of the relevant feature in memory. This points to a memory mechanism, distinct from encoding and retrieval mechanisms involved in agreement, that is likely to be involved in feature assignment, namely, one responsible for temporary storage and maintenance. Memory-based theories of processing locality such as Dependency Locality Theory (DLT, Gibson, 1998, 2000) capture the link between linear distance and processing cost quite well.^3^ According to DLT, integration of a predicted item into the structure currently built incurs a processing cost that is a function of the dependency length measured by the number of intervening lexical items (more precisely, in terms of new discourse referents: each new discourse referents counts as a unit of cost, the total cost is a sum of these units). In the original formulation of DLT, only nouns and verbs count as new discourse referents as designating objects or events (as these works did not discuss dependencies involving adjectives at all); adjectives are not included. Indeed, under the standard assumptions of formal semantics, adjectives fall into the same class as common nouns as both denote properties of individuals or functions from individuals to truth values (cf. Heim and Kratzer, 1998; Delmonte, 2011). We therefore see no principal objection for including adjectives in the list of items that count for the processing cost metric under the general DLT umbrella.

Under this additional assumption, the results of our study suggest that DLT units are good predictors for erroneous productions in feature assignment, but not for agreement. Indeed, in Experiment 1, the gradient of erroneous productions perfectly aligns with the number of intervening nouns and adjectives between the numeral and the final noun: the least errors in the Short condition with 1 DLT unit - an adjective, followed by more errors in the Medial condition with 2 DLT units, an adjective and a noun and the most errors in the Long condition with 3 DLT units, that is, 2 adjectivals and 1 noun (Table 1). In Experiment 2 as well as Experiment 3 showing a similar ratio of errors in the two structural conditions, the number of linear DLT units in all tested conditions are the same, namely, three (Tables 2, 3). Similarly, the results of Stateva and Stepanov's (2013, 2016) corpus and experimental studies of the Bulgarian numeral phrase that established a correlation between the erroneous productions and number of intervening adjectival modifiers between zero and three also naturally fall under the DLT account [cf. (1)/(3)]. The results in Barkalova et al. (2018) study that found no difference in the error ratio between two structurally different conditions, are also predicted by this account: the number of DLT units (nouns and adjectives) in the intervening region in that study are exactly four in both conditions [cf. (6)–(7)].

Furthermore, an account based on a DLT-type linear metric may also explain (i) the apparent divergence of results of the second corpus study in Stateva and Stepanov (2016) which argued against the linear distance approach and (ii) the results of the sentence-completion experiment in Barkalova et al. (2018) (Section 2), which argued against the structural distance approach. Recall that the former study contrasted the pattern of errors when the intervening region between the numeral and the noun consisted either of two adjectives (representing two different syntactic nodes) and an adjective modified by an adverb (representing a single syntactic node), cf. (3a) vs. (5). Recall that this study found more errors in the two adjective condition (3b) than in the single (modified) adjective condition, while the number of lexical items in both conditions was kept constant. On a DLT account, however, this contrast is not surprising: since adverbs do not count as relevant DLT units, the processing cost is actually expected to be greater in the two adjective condition than in the one involving a single adjective. This account is independent of the involvement of the structural condition, consistently with the rest of the results discussed above.

On the other hand, in Experiment 4 testing the agreement relation of nominal concord, the number of units intervening between the agreeing noun and adjective no longer reliably predicts the observed pattern of errors. The agreement/concord process at stake in Experiment 4 involves different, cue-based memory mechanisms relying on a metric whose major component is similarity and not linear proximity. A detailed exploration of the memory mechanisms employed in nominal concord is beyond the scope of this paper. However, it is instructive to pit the results of Experiment 4 against the backdrop of previous studies that experimentally explored agreement processing in this construction type.

### 10.3 Relationship to previous findings on nominal concord

Wagers and McElree (2022) discuss encoding and retrieval mechanisms within NPs involving nominal concord such as that clever risk-taking burglar/^*^burglars and those clever risk-taking burglars/^*^burglar. In English, the only overt agreement reflexes are between the determiner and final noun. Unlike in Slavic (and also other language families, e.g., Romance) intermediate adjectives do not bear agreeing morphological marking. The authors reasoned that, if incoming adjectives that separate the determiner and noun, lead to updating the memory by removing the determiner's number feature (singular or plural) from the immediately accessible memory representation (which they refer to as a point of focal attention) thus necessitating its later retrieval, then the rate at which agreement contrasts are discriminated should slow from the adjacent (no intervening adjectives) to the non-adjacent (intervening adjectives present) conditions. If, on the other hand, the number feature can be maintained in the active memory across the modifiers, then the rates should remain the same across NPs of any length, with or without intervening adjectives. Although the authors do not make it explicit, we believe the idea of maintaining in the active memory across the entire length of a dependency is quite compatible with the idea of temporary storage in the processing locality theories. Using the paradigm of speed-accuracy trade-off, Wagers and McElree (2022) find that processing the agreement relationship between the determiner and noun is actually slowed if the adjacency between the two is disrupted by the adjectives. Thus, according to the authors, the nominal concord is a process rooted in initial encoding and later retrieval of the agreeing (concord) feature and can be disrupted by intervenors. So far the results of Wagers and McElree (2022) are very much in line with our observations regarding the linear distance in both feature assignment as well as nominal concord structures. But there is also an aspect in their findings which is differing from ours in an interesting way. Recall that the linear effect in our Experiment 1 was strictly monotonic across the conditions where the number of intervening adjectives was manipulated: one intervening adjective incurred more errors than no adjective, and two adjectives incurred more errors than one. In Wagers and McElree (2022)'s results, the increase of processing speed with the experimental items involving grammatical contrasts (see above) was not monotonic, rather, the contrast was between the adjacent (no adjectives, faster) and non-adjacent (one or two adjectives, slower) condition. At the same time, there was a robust monotonic effect in the control conditions checking the plausibility of the construction (e.g., *the clever risk-taking jewels*) which were included in the study to control for the potential extra cost of additional modifiers independent of agreement: single and double modification in this case differed in the processing speed substantially. The authors argue that grammatical and plausibility contrasts in their study stem from different sources of processing difficulty: the non-differentiation of the processing speed across the non-adjacent conditions signify simply the displacement of the focus of attention from the determiner, regardless of the actual number of the adjectives outside of it, whereas the monotonic effect in the plausibility NPs signifies the processing cost of adding additional modifiers.

We believe this account harmonizes well with the results of our Experiment 1 as well as the previous studies on the feature assignment in Bulgarian numeral phrase mentioned above: the linear processing cost in Experiment 1 obtains by virtue of adding more modifiers, while the absence of a similar linear effect in Experiment 4 suggests a different mechanism for the nominal concord. Of course, a direct comparison between the nominal concord constructions used in Experiment 4 and Wagers and McElree's English ones is not possible as, strictly speaking, Experiment 4 does not involve an adjacent condition at all (it is curious, however, that we do observe a non-monotonic effect in Experiment 4 as well, with respect to the A0 and A1, as opposed to A2, conditions, but, as discussed in Section 2, it is most likely due to other, unrelated, factors).

### 10.4 Feature assignment and the structural factor

The previous discussion points to one possible source of processing differences between agreement and feature assignment: while the former is couched in the encoding and retrieval procedures, the latter unfolds as a predictive process whereby the prediction is realized as an abstract featural specification for a specific [+count] morphology that is subject to temporary storage. Both processes thus crucially rely on different components of memory to achieve resolution of the respective morphosyntactic dependency. This, however, does not quite explain the fact that agreement but not feature assignment is sensitive to the structural factor. In this part of the discussion we offer some tentative remarks regarding this aspect.

The lack of influence of the structural factor on the rates of feature assignment errors in Bulgarian numeral phrases, as opposed to its established key role in agreement attraction raises an interesting question regarding relevant pertinent characteristic(s) of the real time feature assignment process distinct from those involved in agreement. At least part of a potential answer may come from the incremental character of processing, assuming that it extends also to dependencies inside the noun phrase (cf. Wagers and McElree, 2022 and Section 9.3) and that it can be couched in memory terms. Note that the very notion of retrieval presupposes accessing part of the input already in the memory (in Wagers and McElree, 2022's terms, outside of the focus of attention). Given the incremental character of structure building, computation of the agreement at the retrieval point is bound to refer to the object which is already constructed within a syntactic tree, thus already including information about syntactic structure in the respective memory trace. In contrast, the key point event at the feature assignment takes place not at the tail, as in agreement, but at the head of the dependency, namely, at encoding the abstract prediction for a specific [+count] morphology. At that point, the feature to be assigned actively “awaits” for the input that is not yet there, so that there is no structural information in the memory buffer to begin with. Furthermore, since a feature assignment dependency involves no critical retrievals afterwards, as in the case of agreement, it involves no “backward-looking” aspect that would refer to the previously built structure. Thus, it appears that the predictive or “forward-looking” character of feature assignment suggests no immediate points of reference to structural details, as far as (any potential) retrievals are concerned.

However, incremental structure building also implies structural predictions as is well known from the work on parsing algorithms and strategies (Chomsky and Miller, 1963; Frazier, 1978; Abney and Johnson, 1991; Gibson, 2000; among many others, see Ferreira and Qiu, 2021 for a recent review). For instance, upon encountering the numeral, a typical left-corner parser would activate a phrase structure rule such as NP → Num (AdjP) N, with a prediction for the complement of Num that may include intermediate modifiers (cf. Abney and Johnson, 1991). Temporary storage of the abstract morphological specification of a feature in this environment must a priori be sensitive to the structural information between its licensor (e.g., the numeral) and the recipient. In particular, the system should detect a difference between one and two adjectival modifiers as it incrementally builds the analysis of the incoming material. But the results of our study show that it does not. The question of structural complexity can thus be re-cast in terms of the predictive character of structure building in the time course of processing the NP.

**Figure 14:**
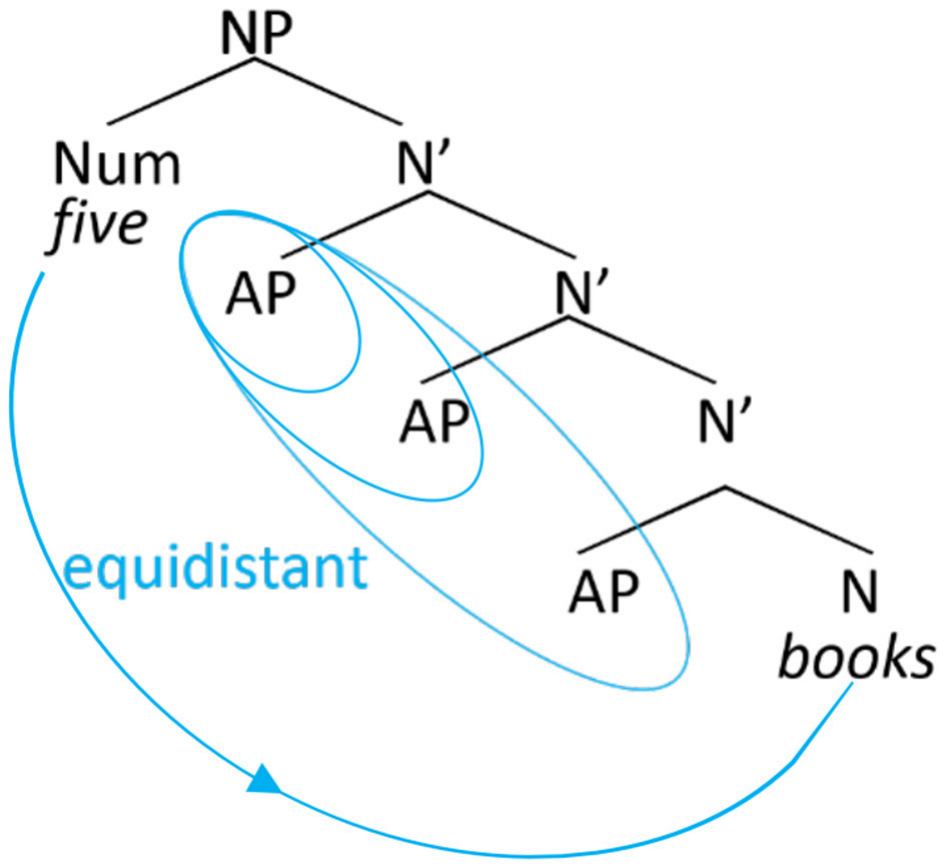
Syntactic structure of the numeral phrase illustrating the equidistance between the numeral and the noun regardless of the number of intervening structural nodes.

We conjecture that in the context of incremental structure building along the lines above, the processor indeed acknowledges structural predictions within the NP along the way. However, when processing the NP-internal feature assignment *dependency* by virtue of maintaining the abstract featural specification in temporary storage, the processor treats both ends of that dependency exactly the same in terms of ease of access, no matter whether they are separated by one or more intervening syntactic nodes [e.g., (6)-(7)]. In fact, there exists a ready syntactic model which entails the absence of the structural effects within smaller structural domains based on a concept of *equidistance* (Chomsky, 1995). In syntax, equidistance means that elements within a phrasal constituent such as NP may become equally accessible to syntactic operations (e.g., constituent displacement), regardless of the structural material that intervenes between them. There are certain structural conditions in order for equidistance to hold among the two ends of a dependency the details of which need not concern us here. For the present purposes it suffices to adopt the syntactic structure of NP in which intervening adjectival modifiers are structural *adjuncts*. Adjuncts are often seen by syntacticians as not adding to the structural complexity of the phrase in the same way as other elements of the phrase (heads, complements and specifiers) do. Under this proviso, in a structure such as *five… books* the numeral *five*, or the feature [+count] that it assigns to the noun (realized as plural in English) can be seen as structurally equidistant to any of the intervening adjectival phrases along the way, no matter how many there are (see Figure 14). As a collateral effect of that, the number of structural nodes intervening between the numeral and the noun (the N'-s in Figure 14) will count as constant regarding structural distance between the numeral and the noun.

Notably, this theoretical approach to equidistance applied in the processing context rests on two fundamental assumptions. One fundamental assumption is architectural: it presupposes a very tight or (close to) transparent connection between the grammatical knowledge and processing routines, the kind of approach that has been taken up in previous studies of agreement attraction (Franck et al., 2006). Second, the account makes a crucial use of the sub-clausal character of the feature assigning dependency which is restricted to a single noun (or numeral) phrase. It would not work for clausal dependencies that span across several syntactic constituents. In addition, this syntactic model makes no prediction about the linear distance, the number of actual intervening lexical items, which is subject to other, non-structural constraints such as those we have seen above. If this much is on the right track, this suggests a potentially far-reaching and testable conclusion that the size of the processing domain plays a key role when accessing processing difficulty/cost in terms of syntactic structure and/or hierarchy.

Naturally, the equidistance account should apply to the nominal concord structures as well. It is difficult to see whether Wagers and McElree's results could be re-interpreted in structural terms as they manipulated the number of adjectives only (which, as we saw above, can be measured either in linear or structural terms, Section 2). But the observed non-monotonic effect distinguishing the adjacent (no intervening adjectives) and non-adjacent (one or two intervening adjectives) conditions in the grammatical (non-plausibility related) concord sentences can potentially be interpreted to that effect, if each adjective instantiates its own syntactic node. Once the “focus of attention” is outside the head of the agreement dependency (in their case, the determiner *that* or *those*) it does not matter how many structural nodes intervene between it and the agreement target (final noun). With some simplifications, the differences between the feature assignment and nominal concord (agreement) structures are summarized in Table 5.

**Table 5:** A comparative summary of findings concerning the linear and structural effects and interference in feature assignment constructions and nominal agreement (concord).

	**Linear effect**	**Interference**	**Structural effect**
Feature assignment, Bulgarian numeral phrase	+	–	–
Agreement/nominal concord, Bulgarian NP with an embedded numeral phrase	–	+	?
Agreement/Nominal concord, English determiner phrase (Wagers and McElree, 2022)	– (non-monotonicity)	?	possibly “–” (non-monotonicity)
Clausal agreement	+	+	+

## 11 Conclusion

This article has provided evidence that feature assignment in the Bulgarian numeral phrase is a process different from agreement (both subject-verb agreement and adjective-noun agreement or nominal concord). Clausal agreement may lead to a faulty retrieval of feature specifications of the intervener to the target and thus display feature mismatch/interference effects. Those retrievals are dependent on the syntactic relation between the intervener and the target. That means that agreement could be accounted for through production feature transmission accounts which allow features from the intervener to be transmitted to the target. All of these accounts assume that agreement is dependent on the syntactic position of the intervener in relation to the target.

On the other hand, feature assignment in the Bulgarian numeral phrase does not lead to interference effects due intervening features and is also not affected by the syntactic complexity between the numeral and the final noun. Instead, the abstract feature specification for the [+count] morphology is encoded and temporarily stored in memory until the suitable host for that feature arrives in the input. The error rates in feature assignment are subject to linear complexity: they increase with a greater number of intervening adjectives and nouns. Memory models that use the linear metric of processing complexity such as DLT account for the linear distance effects. Nominal concord, on the other hand, tends to pattern with more familiar clausal agreement configurations. The size of the processing domain is likely to play a key role in determining the degree of involvement of structural factors in processing featural dependency: smaller domains such as NPs may forfeit structural complexity effects while clausal domains may not.

One limitation of the present study is its scope being limited to the numeral phrase and the [+count] feature. At this point it is not yet clear whether other instances of feature assignment such as case assignment by a verb, preposition, adjective or some functional head in the clausal base will manifest the same processing properties as is the case of feature assignment by a numeral. In other words, it is not yet clear whether the linear effect observed in Experiment 1 and the lack of a structural and feature mismatch effect in Experiments 2 and 3 are due to the properties of the numeral (e.g., whether only numerals involve the temporary storage component of the memory and block feature transmissions from the intervener). In order to address this limitation, further research into other forms of feature assignment outside the numeral phrase will be necessary, like case assignment.

A second limitation of the current study is that we have not tested whether the errors in nominal concord (as observed in Experiment 4) may also be subject to a structural distance effect. In the light of the agreement attraction literature, that would constitute a valid prediction for a future experimental study.

Further investigations should also explore the suitability of models of processing complexity other than DLT-based as a source of potential metric of dependency length in feature assignment, such as, for instance, the memory decay component within a cognitive architecture such as ACT-R (Lewis and Vasishth, 2005; Lewis et al., 2006) or information theoretic principles underlying minimization of length of syntactic dependencies in language use (cf. Hawkins, 1994; Liu et al., 2017). It is possible that the actual metric is not exactly a linear function and also dependent on factors not taken into account in the present work such as time that has passed between the production of the numeral and the target noun (as predicted by the Memory Decay component of ACT-R) or considerations of information compression favoring minimization of syntactic dependencies. For instance, if the memory decay component of ACT-R (also) drives the erroneous productions, we would expect erroneous productions to increase as long as the time between the production of the target noun and the numeral increases even if no semantic material is added between them. This could be tested by manipulating the presence or absence of lexical interveners between the numeral and the target noun while controlling for the temporal interval between them.

The present study is part of a larger research agenda exploring fine distinctions between different types of featural interaction within a sentence that were noted in syntactic theories long ago but until now remained unexplored from the processing side. The focus on features as basic building blocks of sentence composition as well as corresponding memory processes at the sub-clausal (phrasal) level from both perspectives helps refine the respective models and bring them in closer contact aiming for a comprehensive, coherent and balanced picture of the mechanisms and constraints that underlie the knowledge and use of syntactic dependencies.

## Data Availability

The raw data supporting the conclusions of this article will be made available by the authors, without undue reservation.

## Footnotes

^1^To be sure, agreement attraction is also sensitive to the linear proximity between the agreeing constituents. For instance, Franck et al. (2006) found that linear proximity of the attracting noun to the verb in the sentences with pre-verbal subjects leads to greater error ratio than in those with postverbal subjects, in Italian. We discuss linear distance below.

^2^Whether feature assignment is based on the encoding-and-retrieval mechanism at all, similarly to agreement, is a valid empirical question. Our ongoing work suggests that the answer may be affirmative but with some important differences as to what is being encoded and retrieved (see below and Stepanov to appear).

^3^DLT is stated as a syntactic structure- oriented theory since the distance is measured between syntactic heads. However, the simple counting metric in terms of number of intervening new discourse referents suggests a strong linear component to this theory.
