# Supplementary material for: Are feature assignment errors due to attraction? The case of Bulgarian numeral phrase
Source: Front Psychol. 2025 Jul 14;16:1560012. doi: 10.3389/fpsyg.2025.1560012 (PMC12303052; doi:10.3389/fpsyg.2025.1560012)
Supplement: Supplementary file 1 [file Data_Sheet_1.pdf]

Appendix A: Error rate fluctuations by gender of intervening nouns in Experiments 1, 2 and 4

Experiment 1

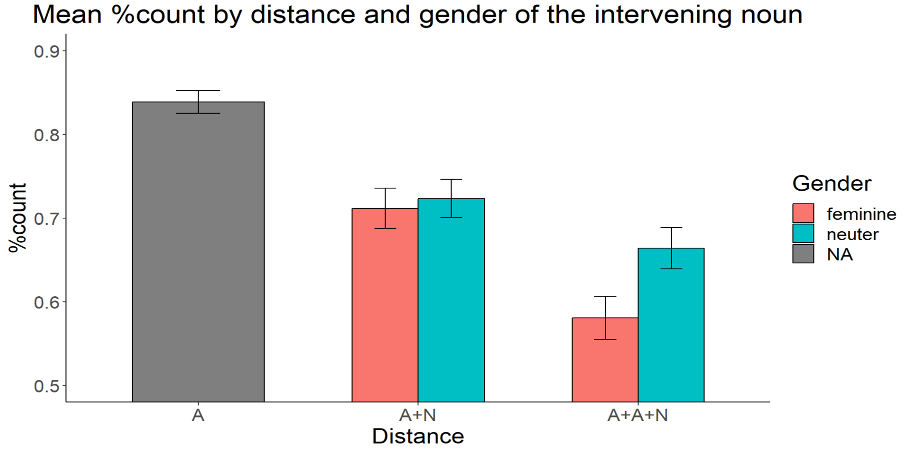

Experiment 2:

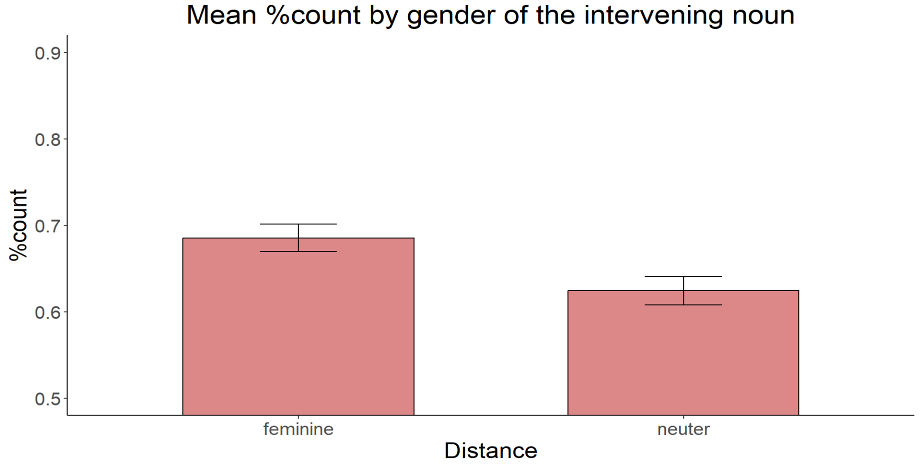

Experiment 4:

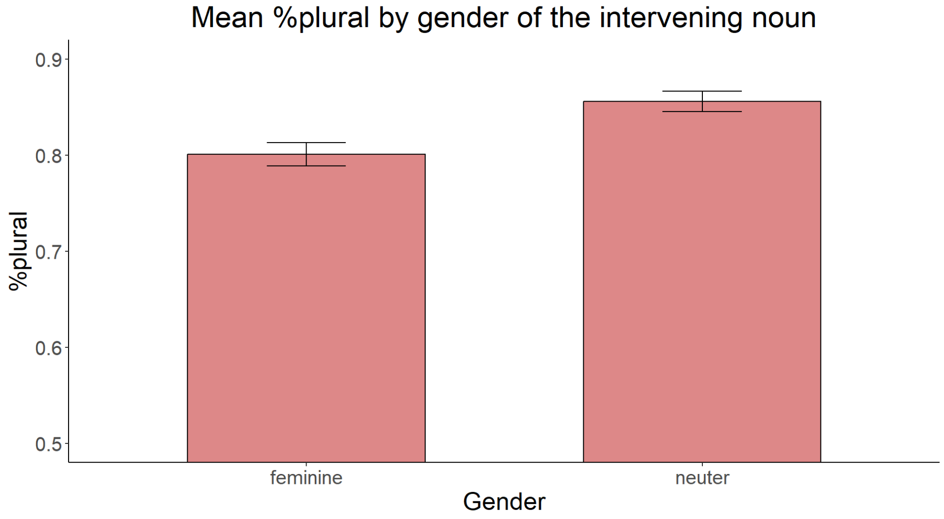

Appendix B: Filler scheme for Experiment 3

(Structure of Experimental conditions (for reference): **[Num [AdjP] [AdjP] Noun-count]**)

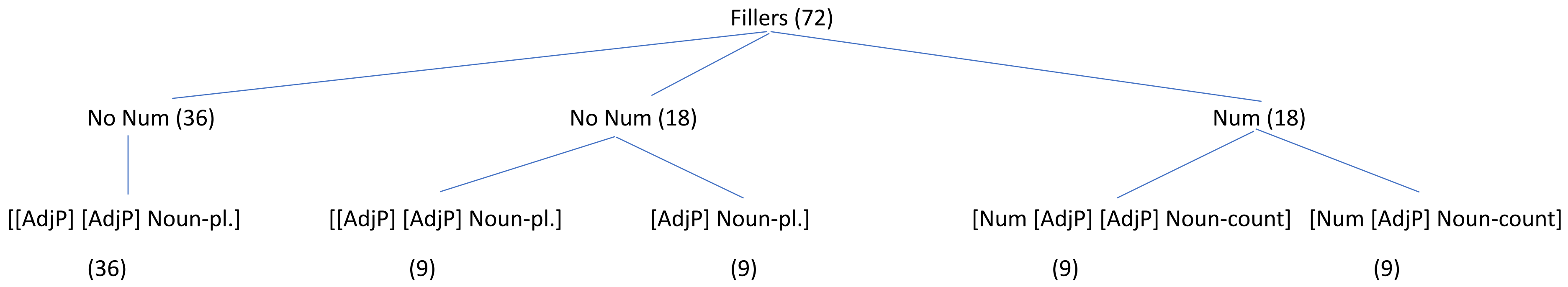

Table A1. Examples of fillers for Condition 1 of Experiment 4 .

Example:	
Preamble:	Ekip-ɤt fotografir-a<br>crew;M-DEF.SHORT;M;SG photograph-PFV;AOR;3;SG;PST
Ending:	ʧern-i-te uplaʃen-i ot meʧoʦ-i-te siniger-i<br>black-PL-PL;DEF scared-PL by male.bear;M-PL-PL;DEF tit-PL<br>'[PREAMBLE The crew photographed] the black tits scared by the male bears.'
Preamble:	Uʧitelʲ-ɤt pokazva-ʃe<br>teacher;M-DEF.FULL;M;S show;IMPFV.IMPF.3.PL.PST
Ending:	dva izvestn-i presiʧaʃt-i ʃtat-i-te rezervat-a<br>two famous-PL crossing-PL state;M-PL-PL;DEF reserve;M-M;COUNT<br>'[PREAMBLE The teacher was showing] two famous reserves crossing the states.'
Preamble:	Sɤprug-a-ta hares-a<br>wife;F-F;SG-DEF;F;SG liked-PFV;AOR;3;SG;PST
Ending:	tri kupen-i ot magazin-i-te prɤsten-a<br>three bought-PL from shop;M-PL-PL;DEF ring;M-M;COUNT<br>'[PREAMBLE The wife liked] three rings bought from the shops.'
